# An efficient and reusable nano catalyst for the synthesis of benzoxanthene and chromene derivatives

**DOI:** 10.1038/s41598-019-40431-x

**Published:** 2019-03-05

**Authors:** Javad Balou, Mohammad A. Khalilzadeh, Daryoush Zareyee

**Affiliations:** Department of Chemistry, Qaemshahr Branch, Islamic Azad University, Qaemshahr, Iran

## Abstract

Potassium fluoride impregnated on clinoptilolite nanoparticles (KF/CP NPs) was investigated as an efficient solid base catalyst for multicomponent condensation reaction. A broad range of aromatic aldehydes and enolizable compounds were condensed with 2-naphthol or 4-hydroxycumarine. The reaction was carried out under a solvent-free condition to give the corresponding benzoxanthene and pyrano [3,2-c]chromene derivatives in high yields. The low cost and availability of catalyst, novel and green procedure makes this strategy more useful for the preparation of tetrahydrobenzo[a]-xanthene-11-one and pyrano [3,2-c]chromene derivatives.

## Introduction

One-pot cascade reactions, multicomponent reactions (MCRs), have attracted much attention over the past decade due to their conciseness and high efficiency^1–3^. MCRs provide a powerful and efficient approach to the synthesis of various heterocyclic compounds which mimic some natural heterocyclic compounds such as xanthenes and benzoxanthenones. The diverse biological properties of xanthenes and benzoxanthenones, such as antiviral^4^, antibacterial and anti-inflammatory activities^5^ are well documented. They have been used in photodynamic therapy^6^, and in some cases, as antagonists for inhibiting the action of zoxalamine. In addition, their derivatives have uses in laser technologies^7^ and as dyes^8^, pH sensitive fluorescent materials for the visualization of biomolecular assemblies^9^. Thus, a multifaceted application has made xanthenes useful synthetic candidates, thereby accentuating the need to develop efficient routes for scaffold manipulation of xanthenes derivatives. The well-known synthetic route toward xanthenes derivatives is the one-pot multi-component condensation reaction between β-naphthol, dimedone and arylaldehydes. Ceric ammonium nitrate^10^, proline triflate^11^, NaHSO_4_.SiO_2_^12^, strontium triflate^13^, Zr(HSO_4_)_4_^14^, dodecatungstophosphoric acid^15^, iodine^16^, InCl_3_/P_2_O_5_^17^ and p-toluenesulfonic acid/ionic liquid ([bmim]BF_4_)^18^, RuCl_4_^19^, brønsted ionic liquid^20^, molecular iodine^21^, trichloroacetic acid^22^, sulfonic acid functionalized imidazolium salts^23^, imidazole or isoquinoline^24^, orange peel^25^, rice husk^26^ and TBAF^27^ are employed as catalysts to promote this reaction.

Similarly, chromenes have also received attention due to their biological activity and their presence in a variety of natural products^28,29^. Pyrano [3,2-c]chromene derivatives, in particular, are a class of heterocycles with a wide range of biological properties^30^ such as spasmolytic, diuretic, anticoagulant, anti-cancer, and anti-anaphylactic activity^31^. Moreover they can be used as cognitive enhancers, for the treatment of neurodegenerative diseases, including Alzheimer’s disease, amyotrophic lateral sclerosis, Parkinson’s disease, Huntington’s disease, AIDS associated dementia and Down’s syndrome as well as for the treatment of schizophrenia and myoclonus^32^. Consequently, a number of synthetic strategies for the construction pyrano[3,2-c]chromene derivatives have been reported^33–36^. Some of the reported procedures, however, require long reaction times, multi-step reactions, complex synthetic pathways, and affordable products with modest yields^37–43^. In addition, the uses of toxic and expensive transition metals and volatile organic solvents limit the scope of this kind of reaction. Therefore, the development of efficient and environmentally benign methods using scalable low-cost materials for the synthesis of these precious compounds^44–47^ is still needed.

Clinoptilolite is abundantly available in many regions around the world. The low cost and special features of clinoptilolite make it valuable in many applications. Clinoptilolite, as a natural zeolite with a high internal surface area, is much more effective because of its high exchange capability for cations particularly for K^+^, allowing more free fluoride anions to function as an effective base.

**Figure 1:**
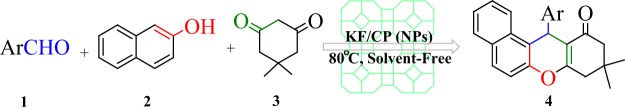
KF/CP NPs catalyzed synthesis of benzo[α]xanthenes.

The use of heterogeneous base catalysts derived from natural zeolite clinoptilolite was investigated for various reactions. It has been proved that this solid base catalyst is able to increase the basicity of fluoride anion in coupling, reactions^48–50^. Since one-pot multi-steps protocols for the effective reduction of costly labor and waste production with the use of unconventional reaction media such as water and also solvent-free condition and recovery and re-use of both catalyst and reaction medium are very important issue, so there is a lot of interest for new applications of potassium fluoride impregnated on zeolites and clays, as a new natural and inexpensive solid base system^51–63^. The high efficiency of this system prompted us to use KF/CP NPs in three component reactions of aldehydes 1, 2-naphthol **2** and dimedone **3** under solvent-free conditions (Fig. 1).

## Results and Discussion

**Figure 2:**
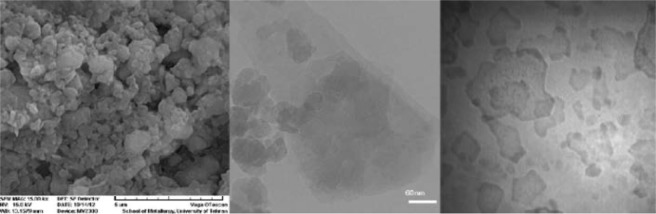
SEM (**a**) and TEM (**b**) images of CP NPs. TEM image of KF/CP NPs (**c**).

The preparation of potassium fluoride impregnated clinoptilolite (KF/CP) is simple without the need for any pre-activation^64,65^. Since nanoparticles have larger surface area available for interactions, which enhances their activity than the large sized particles; in order to improve the efficiency of our catalyst system we used clinoptilolite nanoparticles which could be easily prepared by grinding in a planetary ball mill using a zirconia vial set in dry conditions with a time period of about 20 minutes. The KF/CP (NPs) catalyst was then prepared according to the previously reported procedure^49^. SEM and TEM have analyzed the particle size and morphology of CP NPs and KF@CP NPs samples. The SEM images showed polydisperse morphology and a wide range of particle size distributions (Fig. 2). These features are influenced by the milling process and are thus subject to variation. Although, the TEM images showed some particles in micro-dimensions, the majority of particles are formed in smaller spherical shapes in about 35–65 nm size range. The TEM images of the KF@CP NPs samples show the KF@CP NPs are rather dispersed relative to CP NPs. This could be explained by the adsorption of potassium fluoride onto the CP NPs that increase the negative charges resulted from free fluoride anions on the surface of zeolite leading to the electrostatic repulsion between the layers.

**Figure 3:**
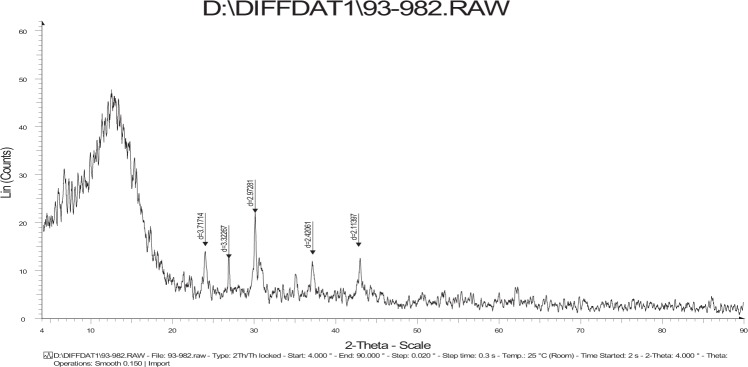
XRD spectra of nano KF/CP.

Figure 3 shows the typical XRD pattern of the prepared nano-zeolite particles that indicates intensity ratio of the major peak observed at 2θ equal to 30.38°. The average crystallite size (L) was calculated from the excess of width line of the diffraction peak in radians (β), Bragg angle in degrees (θ), and Debye-Sherrer’s equation; L = 0.89λ/βcosθ; β is the FWHF of the diffraction peak and λ is the wavelength. The average crystallite sizes of prepared nanozeolite KF/NCP is calculated to be around 69.77 nm.

In continuation of our ongoing research on the applications of heterogeneous catalysts in organic transformations, we planned to utilize KF/CP NPs in the present work as a promising candidate to achieve the condensation of naphthols, aldehydes with dimedone (Scheme 2). In the line of green process development, we first performed the reaction under solvent-free conditions. Fortunately, the reaction proceeded smoothly to produce the desired product using 10% of KF/CP NPs at 80 °C under solvent-free condition. Next, we screened other parameters, such as temperature, quantity and effect of catalyst to find out the best conditions (Table 1).

**Table 1:** Optimization of reaction conditions using benzaldehyde, dimedone and 2-naphthol at 80 °C^66^.


Entry^[a]^	Base/amount (g)^[b]^	Time (h)	Yield (%)^[c]^
1	None	1	—
2	CP/0.02	2	55
3	CP NPs/0.02	2	58
4	KF/0.002	2	70
5	KF/CP NPs/0.02[6%]	2	82
6	KF/CP NPs/0.033[10%]	1	91
7	KF/CP/0.033[15%]	2	82
8	KF/CP NPs/0.05[15%]	1	92
9^d^	KF/CP NPs/0.033[10%]	1	88
10^e^	KF/CP NPs/0.066[20%]	0.8	91

^a^2 mmol of all starting materials were used. ^b^The number in bracket refer to molar percentages of KF. ^c^Isolated yield. ^d,e^The reactions were conducted at 70 and 90 °C, respectively.

It should be noted that the reaction did not yield any product in the absence of KF/CP NPs, confirming the critical role of catalyst in this reaction (Table 1, entry 1). In the absence of potassium fluoride, **4a** was obtained in moderate yield using the same reaction conditions (Table 1, entries 2 and 3). It is also important to mention that when the reaction was performed in the presence of KF and in the absence CP NPs, 70% of desired product was obtained after 2 h (Table 1, entry 4). However, by employing 0.033 g of KF/CP NPs (containing 10 mol% of KF), the reaction yield raised to 91% after 1 hour reaction (Table 1, entry 6). The efficiency of KF/CP NPs is obvious when it is compared to KF/CP with lower yields and longer reaction times even by using higher amounts of catalyst (Table 1, entry 7). The yield of reaction was almost unchanged by enhancing the base, while decreasing the loading of the base to 0.02 g gave **4a** in 82% yield (Table 1, entries 8 and 5). Reduction in temperature to 70 °C led to a slight decrease in the yield (Table 1, entry 9), while further increasing the temperature did not cause a significant improvement on the reaction (Table 1, entry 10).

With the optimized conditions, we examined this reaction with a variety of substituted aromatic aldehydes. The results are summarized in Table 2. A diverse set of aromatic aldehydes with electron-donating and electron-withdrawing substituent as well as hetero aromatic aldehydes were reacted, successfully resulting in good to excellent yields. It was observed that substituents in the aromatic ring of aldehydes have a minor effect on the outcome of the reaction. Aromatic aldehydes with electron withdrawing groups reacted faster than those with electron-donating groups.

**Table 2:** KF/CP NPs catalyzed preparation of xanthenes under solvent-free conditions^a^.


Entry^[a]^	Aldehyde	Product	Time (min)	Yield (%)^[b]^
1	H	**4a**	60	91^67^
2	*o*-Cl	**4b**	60	88^67^
3	*m*-Cl	**4c**	60	90^67^
4	*p*-Cl	**4d**	60	92^67^
5	*m*-NO_2_	**4e**	60	88^67^
6	*p*-NO_2_	**4f**	60	89^67^
7	*p*-CH_3_	**4g**	90	90^67^
8	*p*-OH	**4h**	60	90^67^
9	2-Thienyl	**4i**	60	92

^a^All reactions were run with **1** (2.0 mmol), **2** (2.0 mmol), and **3** (2.0 mmol) in the presence of KF/CP NPs (0.033 g) at 80 °C. ^b^Yields refer to pure products after silica gel column chromatography.

**Figure 4:**
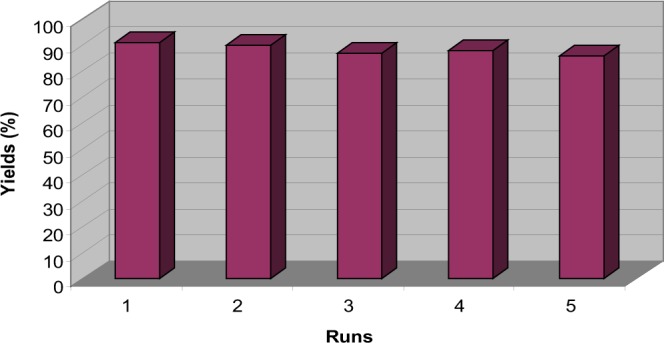
Recycling experiment of KF/CP NPs.

To probe the reusability of the catalyst, the KF/CP NPs, obtained after filtration from the previous reaction was thoroughly washed with dry ethyl acetate, dried under vacuum and reused for the condensation of benzaldehyde, dimedone and 2-naphthol. It is interesting to note that the reaction afforded the product 4a in 90% yield. The recyclability of the catalyst was further confirmed when it was found to exhibit good activity even after the four run with no major decrease in the yield (Fig. 4). For better investigation of catalyst recyclability, we performed two new sets of experiment including both the performing the scaled up reaction and the reusability of catalyst in the middle of reaction. In this context, loading of catalyst decreased to 0.016 g in the reaction of benzaldehyde (2.0 mmol), dimedone (2.0 mmol) and 2-naphthol (2.0 mmol). The yield of corresponding product reached to 30% after 35 min under new reaction conditions. Interestingly, the catalyst was reused again in four successive runs with no significant decreases in its efficiency, providing almost same yields (30%) of the respective product. Besides, when the scale of reaction increased, (benzaldehyde (30.0 mmol), dimedone (30.0 mmol), 2-naphthol (30.0 mmol) and KF/CP NPs (0.495 g), almost the same results obtained in the reusability experiment. These two experiments prove the good recyclability of catalyst during the reaction.

As mentioned already, contrary to the numerous reports on acid catalyzed reactions, there are only few examples on base mediated reactions. In comparison, we should note that the KF/CP catalyst is much cheaper than TBAF that is also susceptible to Hofmann elimination. The same authors reported that the use of KF itself as catalyst under aqueous media was apparently inefficient while KF/CP afforded high yields of corresponding products. In case of organocatalyzed reactions (imidazole or isoquinoline) the catalysts are not recovered and produce large amounts of organic waste. Additionally, this protocol is comparable or superior in some cases than the use of HY zeolite for the synthesis of benzoxanthenones. However, Clinoptilolite is much cheaper than HY zeolite and the resulting KF/CP NPs showed a relatively higher recyclability compared to HY zeolite^45^. On the other hand, combination of potassium fluoride and Clinoptilolite provides a strong solid base catalyst which is comparable to the more expensive TBAF^27^ and trityl chloride (TrCl)^44^, resulting the desired products in much shorter reaction times.

**Figure 5:**
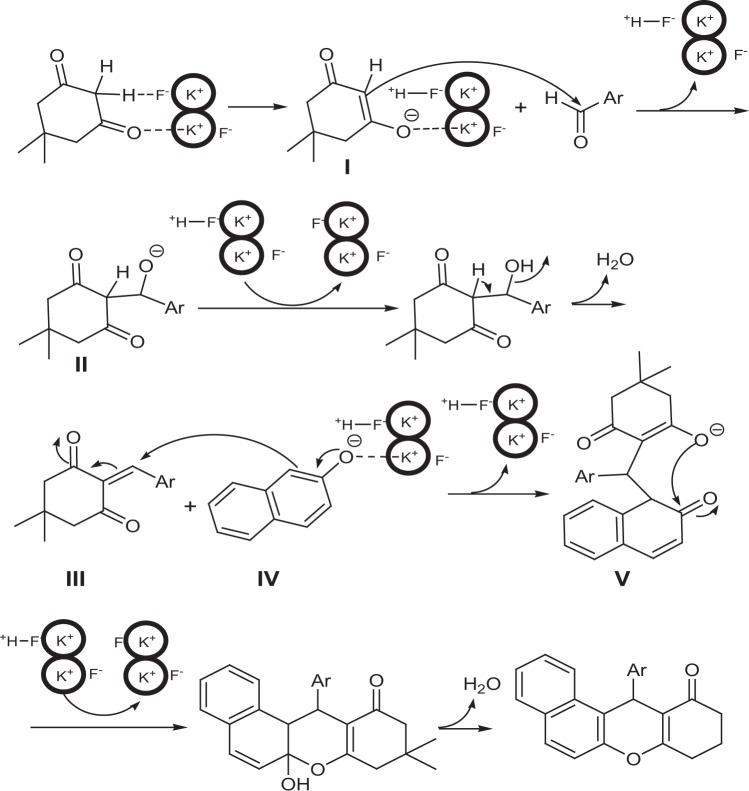
Proposed mechanism for the condensation reaction of aldehydes, 2-naphthol, and dimedone.

The plausible mechanism is indicated in Fig. 5. The reaction most likely starts with the activation of acidic α-hydrogens via the abstraction of α-H by the fluorine anion of potassium fluoride impregnated on CP. The carboanion I attacks to carbonyl group of aldehyde and afford intermediate II that undergoes protonation and dehydration using solid base to give compound III. The same activation occurs for β-naphthol using solid base to generate the corresponding anion IV. The Michael addition of anion IV to compound III produces intermediate V. Finally, by protonation and removal of water from V, the final product is produced.

**Figure 6:**
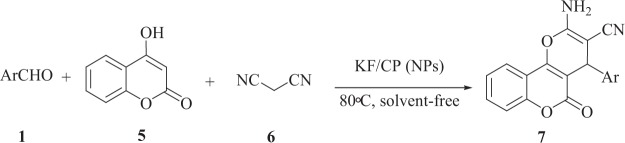
KF/CP NPs catalyzed synthesis of Pyrano [3,2-c]chromene derivatives **7**.

To expand the scope of this method, 4-hydroxycumarine and malononitrilewere used as enolizable C-H activated compounds instead of dimedone under the same conditions (Fig. 6)^66^.

Table 3 shows that in all cases the reactions afforded the corresponding products with good yields.

**Table 3:** KF/CP NPs catalyzed synthesis of pyranochromenes.

Entry^[a]^	Aldehyde	Product	Time (min)	Yield (%)^[b]^ ^66,68,69^
1	C_6_H_5_-	**7a**	60	85
2	*p*-Br-C_6_H_4_-	**7b**	65	90
3	*p*-Cl-C_6_H_4_-	**7c**	65	93
4	*p*-CN-C_6_H_4_-	**7d**	70	95
5	2,3-Cl_2_-C_6_H_3_-	**7e**	70	90
6	2,4-Cl_2_-C_6_H_3_-	**7f**	60	87
7	2,6-Cl_2_-C_6_H_3_-	**7g**	60	85

^a^All reactions were run with **1** (2.0 mmol), **5** (2.0 mmol), and **6** (2.0 mmol) in the presence of KF/CP NPs (0.033 g) at 80 °C. ^b^Yields refer to pure products after silica gel column chromatography.

**Figure 7:**
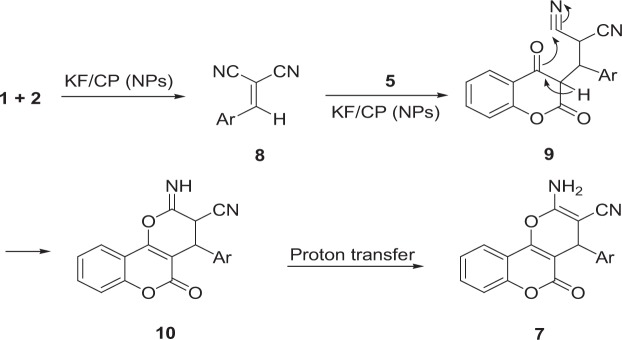
Proposed mechanism for the synthesis of pyranochromenes derivatives.

A tentative mechanism for this transformation is proposed in Fig. 7. It is conceivable that the initial event is the formation of **8** which undergoes Knoevenagel condensation with malononitrile. This intermediate is subsequently attacked by 4-hydroxycoumarin to generate **9**. Intermediate **9** undergoes cyclization to produce **7**.

Under similar conditions, three component reactions of aldehydes 1, 2-naphthol derivatives **11** and dimedone **3** in the presence of KF/CP NPs produce chromene derivatives **12** in good yields (Table 4).

**Table 4:** KF/CP NPs catalyzed synthesis of benzo[α]xanthenes.


Entry^[a]^	Aldehyde	Naphthol	Product	Time (min)	Yield (%)^[b]^
1	4-Cl-C_6_H_4_-	5-nitro-2-naphthol	**12a**	72	92
2	3-OH-C_6_H_4_-	6-methoxy-2-naphthol	**12b**	85	90

^a^All reactions were run with **1** (2.0 mmol), **11** (2.0 mmol), and **3** (2.0 mmol) in the presence of KF/CP NPs (0.033 g) at 80 °C. ^b^Yields refer to pure products after silica gel column chromatography.

To expand the scope of this method, 4-hydroxycumarine and ethyl 2-cyanoacetate were used as enolizable C-H activated compounds instead of malononitrile under the same conditions (Table 5). In this condition, pyranochromenes and lactones are produced. The spectral data of the related products confirmed this claim. Table 5 shows that in all cases the reactions proceeded well to afford the corresponding products with good yields.

**Table 5:** KF/CP NPs catalyzed synthesis of pyranochromenes and lactones.


Entry^[a]^	Aldehyde	R	Product	Time (min)	Yield (%)^[b]^
**1**	C_6_H_5_-	7-Br (a)	**14a and 15a**	**54**	**55 and 43**
**2**	*p*-Br-C_6_H_4_-	6-MeO (b)	**14b and 15b**	**50**	**58 and 37**

^a^All reactions were run with **1** (2.0 mmol), **5** (2.0 mmol), and **13** (2.0 mmol) in the presence of KF/CP NPs (0.033 g) at 80 °C. ^b^Yields refer to pure products after silica gel column chromatography.

## Conclusions

In summary, we developed an easy process to produce benzoxanthenes and pyranochromenes in good to excellent yields using a cheap solid base catalyst in one-pot three-component reaction. Moreover, the catalyst could be readily separated without significant loss of catalytic activity after three runs. The simple experimental procedure both for preparation of catalyst and products, short reaction times, good yields, tolerability of various functional groups and low cost of the catalyst are the main advantages of the present method.

## Experimental

### General

All chemicals used in this work were purchased from Fluka (Buchs, Switzerland) and were used without further purification. Clinoptilolite was obtained from Afrand tooska Company in the region of Semnan. Elemental analyses for C, H, and N were performed using a Heraeus CHN–O-Rapid analyzer. Mass spectra were recorded on a FINNIGAN-MAT 8430 spectrometer operating at an ionization potential of 70 eV. IR spectra were measured on a Shimadzu IR-460 spectrometer. The morphology of nanoparticles of KF/Clinoptilolite was characterized by scanning electron microscopy (SEM) using a Holland Philips XL30 microscope. Crystalline structure of KF/CP (NPs) was characterized by X-ray diffraction (XRD) analysis at room temperature using a Holland Philips Xpert X-ray powder diffractometer, with CuKα radiation (λ = 0.15406 nm), with 2θ ranging from 20 to 80°^67^. The average crystallite size was calculated using Scherrer’s formula; D = 0.9λ/β cosθ^67^, where D is the diameter of the nano particles, λ (CuKα) = 1.5406 Å and βisthe full-width at half-maximum of the diffraction lines^66^. ^1^H, and ^13^C NMR spectra were measured with a BRUKER DRX-500 AVANCE spectrometer at 500.1 and 125.8 MHz, respectively. ^1^H and ^13^C spectra were obtained for solutions in CDCl_3_ using TMS as internal standard or 85% H_3_PO_4_ as external standard.

### Preparation of KF/CP NPs

Nano sized natural Clinoptilolite zeolite was prepared by grinding in a planetary ball mill using a zirconia vial set in dry conditions with a time of about 20 min. Then, the KF/CP (NPs) catalyst was prepared according to previously reported procedure^50^. Thus, 1 g of KF was dissolved in distilled water (10 ml) and nano Clinoptilolite (9 g). The mixture was stirred for 50 min. Then, the water was evaporated at 60–70 °C under reduced pressure. Moreover, the impregnated Clinoptilolite was dried at 70–80 °C in a vacuum drying oven for 30 h. The resulting material was powdered using a pestle and mortar. The obtained KF/CP (NPs) was stored in a desiccator until required.

### General procedure for the synthesis of 4 and 7

A mixture of aldehyde (2 mmol), 2-naphthol or 4-hydroxycumarine (2 mmol), dimedone or malononitrile (2 mmol) and KF/CP NPs (0.033 g) was stirred at 80 °C in an oil bath for 60–120 min, as indicated by TLC for a complete reaction. Upon completion, dried ethyl acetate was added and the catalyst was separated by filtration from the product solution, washed with ethyl acetate, and used for subsequent cycles after drying under vacuum. The solvent of residue was removed under reduced pressure. The crude product was purified by column chromatography on silica gel using ethyl acetate/hexane as the eluent or recrystalization in ethanol. The products were characterized using spectral data and comparison of their physical data with the literature.

### General procedure for the synthesis of 12, 14 and 15

A mixture of aldehyde (2 mmol), 2-naphthol derivatives or 4-hydroxycumarine (2 mmol), dimedone or ethyl 2-cyanoacetate (2 mmol) and KF/CP NPs (0.033 g) was stirred at 80 °C in an oil bath for 60–120 min, as indicated by TLC for a complete reaction. Upon completion, dried ethyl acetate was added and the catalyst was separated by filtration from the product solution, washed with ethyl acetate, and used for subsequent cycles after drying under vacuum. The solvent of residue was removed under reduced pressure. The crude product was purified by column chromatography on silica gel using ethyl acetate/hexane as the eluent or recrystallization in ethanol. The products were characterized using of spectral data and comparison of their physical data with the literature (see supporting information).

## Supplementary information

Potassium Fluoride Supported on Clinoptilolite Nanoparticles: an Efficient Heterogeneous Catalyst for the Synthesis of Benzoxanthene and Chromene Derivatives
