# Supplementary material for: An efficient and reusable nano catalyst for the synthesis of benzoxanthene and chromene derivatives
Source: Sci Rep. 2019 Mar 5;9:3605. doi: 10.1038/s41598-019-40431-x (PMC6401364; doi:10.1038/s41598-019-40431-x)
Supplement: Supplementary file 1 — Potassium Fluoride Supported on Clinoptilolite Nanoparticles: an Efficient Heterogeneous Catalyst for the Synthesis of Benzoxanthene and Chromene Derivatives [file 41598_2019_40431_MOESM1_ESM.doc]

An efficient and reusable nano catalyst for the synthesis of benzoxanthene and chromene derivatives

Javad Balou, Mohammad A. Khalilzadeh*, Dariush Zareyee

Department of Chemistry, Qaemshahr Branch, Islamic Azad University, Qaemshahr, Iran

Tel: +98 911 113 0400 ; E-mail: [khalilzadeh73@gmail.com](mailto:khalilzadeh73@gmail.com)

Notes

**9,9-Dimethyl-12-phenyl-8,9,10,12-tetrahydro-benzo[a]- xanthen-11-one (4a)**^66^

White crystals; mp 154-155 ^o^C; Yield: (91%). IR (KBr)(*ν*_max_/cm^-1^): 3127, 2956, 1657, 1597, 1395, 1376, 1274, 1167, 1125 cm^-1^. ^1^H NMR (500 MHz, CDCl_3_): δ (ppm) 0.98 (s, 3H), 1.14 (s, 3H), 2.28 (dd, *J* = 7.2 Hz, *J* = 15.8 Hz, 2 H), 2.63 (s, 2 H), 5.75 (s, 1H), 7.12-7.16 (m, 1 H), 7.18-7.23 (m, 2 H), 7.42-7.53 (m, 5H), 7.75-7.82 (m, 2 H),8.02 (d, *J* = 8.5 Hz, 1H). ^13^C NMR (125 MHz, CDCl_3_): δ (ppm) 27.3, 29.4, 32.5, 34.8, 41.6, 51.2, 114.8, 117.6, 118.2, 124.3, 125.3, 126.8 (2 CH), 127.6, 128.5, 128.8, 129.2, 129.7 (2 CH), 131.7, 132.4, 145.2, 148.3, 164.2, 197.4. MS (ESI) m/z 355 (M^+^, 15), 274 (20).

**12-(2-Chlorophenyl)-9,9-dimethyl-8,9,10,12-tetrahydrobenzo [a]xanthen-11-one (4b)**^66^

White solid; m.p. 179–180 ^o^C; Yield: (88%). IR (KBr)(*ν*_max_/cm^-1^): 3076, 2937, 1653, 1378, 1234, 1152 cm-^1^. ^1^H NMR (500 MHz, CDCl_3_): δ (ppm) 1.02 (s, 3 H), 1.15 (s, 3 H), 2.26 (d, *J* = 16.4 Hz, 1 H), 2.37 (d, *J* = 16.4 Hz, 1 H), 2.65 (s, 2 H), 6.04 (s, 1 H), 7.08–7.12 m, 2 H), 7.32–7.52 (m, 5 H), 7.75–7.82 (m, 2 H), 8.23 (d, *J* = 8.4 Hz, 1 H). ^13^C NMR (75 MHz, CDCl_3_): δ (ppm) 27.3, 29.6, 32.6, 33.4, 41.8, 51.2, 114.3, 118.2, 118.8, 124.3, 125.3, 127.4, 128.3, 128.9, 129.2, 129.7, 130.4, 131.8, 132.4, 132.9, 133.3, 143.2, 148.2, 164.5,197.4. Anal. Calcd for C_25_H_21_ClO_2_: C, 77.21; H, 5.44. Found: C, 77.49; H, 5.56.

**12-(3-Chlorophenyl)-9,9-dimethyl-9,10-dihydro-8H-benzo[a] xanthen-11(12H)-one (4c)**^66^

White powder, m.p. 192-194 ^o^C; Yield: (90%). ^1^H NMR (500 MHz, DMSO-d_6_): δ (ppm) 0.88 (s, 3H), 1.03 (s, 3H), 2.11 (d, *J* = 12.8 Hz, 1H), 2.32 (d, *J* = 16.1 Hz, 1H), 2.61 (Distorted AB System, 2H), 5.60 (s, 1H), 7.11 (s, 1H), 7.19 (d, *J* = 4.4 Hz, 2H), 7.36 (s, 1H), 7.40-7.50 (m, 3H), 7.89-8.01 (m, 2H), 8.02 (d, *J* = 8.3 Hz, 1H). ^13^C NMR (125 MHz, DMSO-d_6_): δ (ppm) 27.0, 29.6, 32.7, 34.7, 41.0, 50.9, 113.5, 117.3, 118.0, 124.0, 125.9, 127.2, 127.6, 128.2, 128.8, 129.4, 130.3, 130.9, 131.3, 131.9, 133.5, 148.0, 148.1, 165.0, 196.7.

**12-(4-Chlorophenyl)-9,9-dimethyl-9,10-dihydro-8H-benzo[a] xanthen-11(12H)-one (4d)**^66^ White crystals; mp 181-182 ^o^C. Yield: (92%). IR (KBr)(*ν*_max_/cm^-1^): 3133, 2958, 1648, 1596, 1483, 1400, 1375, 1224, 1139, 1009, 841, 747, 535 cm^-1^. ^1^H NMR (500 MHz, CDCl_3_): δ (ppm) 1.04 (s, 3H), 1.17 (s, 3H), 2.32 (dd, *J* = 7.8 Hz, *J*_2_ = 15.7 Hz, 2 H), 2.63 (s, 2 H), 5.74 (s, 1 H), 7.16-7.23 (m, 2 H), 7.23-7.47 (m, 5 H), 7.75-7.82 (m, 2 H), 7.96 (d, *J* = 8.5 Hz, 1 H). ^13^C NMR (125 MHz, CDCl_3_): δ (ppm) 27.4, 29.6, 32.7, 34.6, 41.6, 51.3, 114.7, 117.6, 124.2, 125.7, 127.6, 128.7 (2 CH), 129.2, 129.7 (2 CH), 130.4 (2 CH), 131.6, 132.3, 132.8, 143.7, 148.2, 164.7, 197.5. MS (EI) *m/z* 388 (M^+^, 15), 221 (20), 277 (100).

**9,9-Dimethyl-12-(3-nitrophenyl)-9,10-dihydro-8H-benzo[a] xanthen-11(12H)-one (4e)**^66^

White crystals; mp 169-170 ^o^C. Yield: (88%). IR (KBr)(*ν*_max_/cm^-1^): 3127, 2957, 2872, 1657, 1586, 1487, 1376, 1247 cm^-1^. ^1^H NMR (500 MHz, DMSO-d_6_): δ (ppm) 0.84 (s, 3H), 1.04 (s, 3H), 2.13 (d, *J* = 16.0 Hz, 1H), 2.33 (d, *J* = 16.1 Hz, 1H), 2.64 (Distorted AB System, 2H), 5.77 (s, 1H), 7.40-7.49 (m, 4H), 7.73 (d, *J* = 7.8 Hz, 1H), 7.89-7.95 (m, 3H), 8.03 (d, *J* = 8.4 Hz, 1H), 8.17 (t, *J* = 1.8 Hz, 1H). ^13^C NMR (125 MHz, DMSO-d_6_): δ (ppm) 27.0, 29.5, 32.7, 34.8, 41.7, 50.8, 113.2, 116.7, 117.0 118.0, 122.3, 123.4, 124.0, 126.0, 128.3, 129.5, 130.6, 131.2, 132.0, 135.6, 147.7, 148.1, 148.4, 165.2, 196.8 ppm. MS (EI) m/z 399 (M^+^, 8), 165 (56), 221 (40), 277 (100).

**12-(4-Nitrophenyl)-9,9-dimethyl-8,9,10,12-tetrahydrobenzo [a]xanthen-11-one (4f)**^66^

White solid; mp 183–185 °C. Yield: (89%). ^1^H NMR (500 MHz, CDCl_3_): δ (ppm) 1.05 (s, 3 H), 1.16 (s, 3 H), 2.27 (d, *J* = 15.4 Hz, 1 H), 2.38 (d, *J* = 15.4 Hz, 1 H), 2.64 (s, 2 H), 7.42 (d, *J* = 7.8 Hz, 1 H), 7.38–7.45 (m, 2 H), 7.57 (d, *J* = 8.2 Hz, 2 H), 7.82–7.87 (m, 3 H), 8.08 (d, *J* = 7.5 Hz, 2 H). ^13^C NMR (125 MHz, CDCl_3_): δ (ppm) 27.6, 30.2, 32.7, 35.6, 42.3, 51.7, 114.3, 116.6, 117.3, 123.7, 124.2, 125.8, 128.2, 129.3, 130.4, 130.8, 131.8, 132.3, 147.2, 148.6, 152.5, 165.4, 197.3.

**9,9-Dimethyl-12-p-tolyl-8,9,10,12-tetrahydrobenzo[a]xanthen-11-one (4g)**^66^

White solid, m.p.175–176°C; Yield: (90%). IR (KBr) (*ν*_max_/cm^-1^): 3015, 2878, 1668, 1636, 1587, 1474, 1378, 1295 cm^–1^. ^1^H NMR (500 MHz, CDCl_3_): δ (ppm) 1.12 (s, 3H), 1.18 (s, 3H), 2.34 and 2.42 (AB system, *J* = 15.4 Hz, 2H), 2.63 (s, 2H), 5.73 (s, 1H), 7.14 (d, *J* = 7.5 Hz, 2H), 7.26–7.42 (m, 4H), 7.53 (d, *J* = 7.8 Hz, 1H), 7.82 (d, *J* = 7.7 Hz, 2H), 8.08 (d, *J* = 7.8 Hz, 1H). ^13^C NMR (125 MHz, CDCl_3_): δ (ppm) 21., 27.6, 30.2, 33.4, 34.8, 41.9, 51.4, 114.7, 117.9, 118.5, 124.3, 125.7, 127.6, 128.9, 129.4, 129.7, 130.6, 131.7, 132.2, 136.3, 142.4, 148.5, 164.2, 197.6. Anal. Calcd. for C_26_H_24_O_2_: C, 84.75; H, 6.57. Found: C, 85.02; H, 6.38.

**12-(4-Hydroxyphenyl)-9,9-dimethyl-8,9,10,12-tetrahydrobenzo[a ]xanthen-11-one (4h)**^66^

White solid; mp 213–214 °C. Yield: (90%). ^1^H NMR (500 MHz, CDCl3): δ (ppm) 1.07 (s, 3 H), 1.16 (s, 3 H), 2.37 (d, *J* = 15.6 Hz, 1 H), 2.42 (d, *J* = 15.7 Hz, 1 H), 2.62 (s, 2 H), 5.68 (s, 1 H), 6.73 (d, *J* = 7.8 Hz, 2 H), 6.82 (s, 1 H), 7.23 (d, *J* = 7.8 Hz, 2 H), 7.36 (d, *J* = 7.8 Hz, 1 H), 7.42 (t, *J* = 7.5 Hz, 1 H), 7.47 (t, *J* = 7.8 Hz, 1 H), 7.78–7.86 (m, 2 H), 8.12 (d, *J* = 8.2 Hz, 1 H). ^13^C NMR (125 MHz, CDCl_3_): δ (ppm) 27.7, 32.6, 29.7, 34.6, 42.4, 51.5, 115.2, 116.3, 117.7, 118.6, 124.2, 125.7, 128.2, 128.8, 129.4, 130.4, 132.3, 132.8, 137.3, 148.4, 155.2, 165.7, 198.5.

**9,9-Dimethyl-12-(thiophen-2-yl)-9,10-dihydro-8H-benzo[a] xanthen-11(12H)-one (4i)**^66^

White crystals; mp 180-181 ^o^C. Yield: (92%). IR (KBr)(*ν*_max_/cm^-1^): 3125, 2968, 1678, 1586, 1378, 1247, 1298 cm^-1^. ^1^H NMR (500 MHz, CDCl_3_): δ (ppm) 1.17 (s, 3H), 1.23 (s, 3H), 2.37 (s, 2H), 2.63 (s, 2H), 6.12 (s, 1H), 6.75-6.82 (m, 2H), 7.12-7.23 (m, 1H), 7.37-7.64 (m, 3H), 7.75-7.83 (m, 2H), 8.14 (d, *J* = 8.5 Hz, 1H). ^13^C NMR (125 MHz, CDCl_3_): δ (ppm) 27.7, 30.2, 30.8, 32.8, 41.9 51.7, 114.3, 117.8, 118.2, 124.3, 124.8, 125.7, 126.3, 126.8, 127.6, 128.5, 129.7, 131.6 (2 CH), 148.2, 149.3, 165.3, 197.3. MS (EI) *m/z* 360 (M^+^, 75), 165 (55), 221 (50), 277 (100), 327 (60).

**2-Amino-5-oxo-4-phenyl-4H,5H-pyrano[3,2-c]chromene-3-carbonitrile (7a)**

White solid, m.p. 255–257 ^o^C. Yield: (85%). IR (KBr) (*ν*_max_/cm^-1^) = 3377, 3285, 3162, 2178, 1722, 1696, 1624 cm^-1^. ^1^H NMR (500 MHz, DMSO-d_6_): δ (ppm) 4.50 (s, 1 H), 7.28 (d, ^3^*J* = 7.8 Hz, 2 H), 7.32 (d, ^3^*J* = 7.8 Hz, 1 H), 7.37 (t, ^3^*J* = 7.5 Hz, 2 H), 7.45 (br s, 2 H), 7.52 (d, ^3^*J* = 8. 4 Hz, 2 H), 7.58 (t, ^3^*J* = 7.6 Hz, 1 H), 7.78 (t, ^3^*J* = 7.5 Hz, 1 H), 7.97 (d, ^3^*J* = 7.8 Hz, 1 H) ppm. ^13^C NMR (125 MHz, DMSO-d_6_): δ (ppm) 59.2, 105.3, 114.3, 118.2, 120.7, 123.8, 126.2, 128.7, 129.3, 129.7, 134.5, 144.8, 153.6, 154.9, 159.4, 161.3. MS (EI, 20 eV): m/z (%) 316 (M^+^, 15), 239 (100). Anal. Calcd for C_19_H_12_N_2_O_3_ (316.31) C, 72.15; H, 3.79; N, 8.86. Found: C, 72.19; H, 3.72; N, 8.83.

**2-Amino-5-oxo-4-(4-bromophenyl)-4H,5H-pyrano[3,2-c] chromene-3-carbonitrile (7b)**

White solid, mp = 258-260 ^o^C. Yield: (90%). IR (KBr) (*ν*_max_/cm^-1^) = 3405, 3357, 3234, 2247, 1728, 1696, 1578, 1278 cm^-1^. ^1^H NMR (500 MHz, DMSO-d_6_): δ (ppm) 4.75 (s, 1 H), 7.52 (d, ^3^*J* = 6.7 Hz, 1 H), 7.56 (t, ^3^*J* = 7.6 Hz, 1 H), 7.64 (br s, 2 H), 7.73 (t, ^3^*J* = 7.6 Hz, 1 H), 7.82 (t, ^3^*J* = 7.5 Hz, 1 H), 7.86 (d, ^3^*J* = 6.8 Hz, 1 H), 8.02 (d, ^3^*J* = 6.8 Hz, 1 H), 8.15 (d, ^3^*J* = 8.4 Hz, 1H), 8.23 (d, ^3^*J* = 7.8 Hz, 1 H). ^13^C NMR (125 MHz, DMSO-d6): δ (ppm) 58.2, 104.2, 114.3, 117.6, 120.7, 123.8, 123.9, 124.2, 126.0, 131.4, 134.2, 136.7, 147.2, 153.6, 155.2, 159.5, 161.7.

**2-Amino-5-oxo-4-(4-chlorophenyl)-4H,5H-pyrano[3,2-c]chromene-3-carbonitrile (7c)**

White solid, m. p. 262-264 ^o^C. Yield: (93%). IR (KBr) (*ν*_max_/cm^-1^) = 3385, 3327, 3287, 2478, 1718, 1686, 1625 cm^-1^. ^1^H-NMR (500 MHz, DMSO-d_6_):δ (ppm) 4.52 (s, 1 H), 7.37 (d, ^3^*J* = 8.2 Hz, 2 H), 7.42 (br s, 2 H), 7.43 (d, ^3^*J* = 8.2 Hz, 2 H), 7.52 (d, ^3^*J* = 8.2 Hz, 1 H), 7.58 (t, ^3^*J* = 7.6 Hz, 1 H), 7.76 (t, ^3^*J* = 7.8 Hz, 2 H), 8.05 (d, ^3^*J* = 7.8 Hz, 1 H). ^13^C NMR (125 MHz, DMSO-d_6_): δ (ppm) 59.2, 104.6, 114.4, 117.8, 120.5, 123.8, 126.2, 129.8, 130.7, 133.4, 134.2, 143.9, 153.8, 154.7, 159.3, 161.8. MS (EI, 20 eV): m/z (%): 350.2 (M^+^, 10), 239 (100). Anal. Calcd for C_19_H_11_N_2_O_3_Cl (350.76) C, 65.05; H, 3.14; N, 7.99. Found: C, 65.17; H, 3.12; N, 7.82%.

**2-Amino-5-oxo-4-(4-cyanophenyl)-4H,5H-pyrano[3,2-c] chromene-3-carbonitrile (7d)**

White solid, mp = 264-266 ^o^C. Yield: (95%). IR (KBr) (*ν*_max_/cm^-1^) = 3365, 3327, 3256, 2478, 1715, 1687, 1626 cm^-1^. ^1^H NMR (500 MHz, DMSO-d_6_): δ (ppm) 4.46 (s, 1 H), 6.93 (d, ^3^*J* = 8.1 Hz, 2 H), 7.25 (d, ^3^*J* = 8.3 Hz, 2 H), 7.45 (br s, 2 H), 7.53 (d, ^3^*J* = 8.3 Hz, 1 H), 7.54 (t, ^3^*J* = 7.8 Hz, 1 H), 7.78 (t, ^3^*J* = 7.7 Hz, 1 H), 7.97 (d, ^3^*J* = 7.7 Hz, 1 H). ^13^C NMR (125 MHz, DMSO-d_6_): δ (ppm) 59.7, 105.6, 114.2, 115.6, 118.4, 120.6, 124.2, 126.4, 130.2, 134.3, 136.8, 153.2, 154.8, 159.6, 159.5, 161.7. Anal. Calcd. For C_20_H_11_N_3_O_3_ (341.32) C, 70.38; H, 3.25; N, 12.31. Found: C, 70.65; H, 3.42; N, 12.46.

**2-Amino-5-oxo-4-(2,3-dichlorophenyl)-4H,5H-pyrano[3,2-c]chromene-3-carbonitrile (7e)**

White solid, mp = 248-250 ^o^C. Yield: (90%). IR (KBr) (*ν*_max_/cm^-1^) = 3456, 3267, 3153, 3064, 2287, 1727, 1667, 1528 cm^-1^. ^1^H NMR (500 MHz, DMSO-d_6_): δ (ppm) 4.92 (s, 1 H), 7.44 (d, ^3^*J* = 7.6 Hz, 1 H), 7.52 (d, ^3^*J* = 7.8 Hz, 1 H), 7.57 (br s, 2 H), 7.63 (d, ^3^*J* = 8.0 Hz, 1 H), 7.64 (t, ^3^*J* = 7.8 Hz, 1 H), 7.72 (d, ^3^*J* = 7.8 Hz, 1 H), 7.82 (t, ^3^*J* = 7.8 Hz, 1 H), 8.04 (d, ^3^*J* = 8.2 Hz, 1 H). ^13^C NMR (125 MHz, DMSO-d_6_): δ (ppm) 57.7, 104.2, 114.6, 118.4, 120.7, 124.5, 126.7, 129.6, 130.2, 133.8, 134.3, 134.8, 135.2, 141.2, 154.1, 156.4, 159.7, 161.8.

**2-Amino-5-oxo-4-(2,4-dichlorophenyl)-4H,5H-pyrano[3,2-c] chromene-3- carbonitrile (7f)**

White solid, m. p. 255-257 ^o^C. Yield: (87%). IR (KBr) (*ν*_max_/cm^-1^) = 3465, 3342, 3254, 3125, 2287, 1718, 1685, 1578cm^-1^. ^1^H NMR (500 MHz, DMSO-d_6_): δ (ppm) 5.02 (s, 1 H), 7.43 (d, ^3^*J* = 8.3 Hz, 1 H), 7.46 (d, ^3^*J* = 8.3 Hz, 1 H), 7.52 (br s, 2 H), 7.63 (d, ^3^*J* = 8.3 Hz, 1 H), 7.72 (t, ^3^*J* = 7.7 Hz, 1 H), 7.78 (d, ^4^*J* = 2.1 Hz, 1 H), 7.83 (t, ^3^*J* = 8.2 Hz, 1 H), 7.95 (d, ^3^*J* = 8.9 Hz, 1 H) ppm. ^13^C NMR (125 MHz, DMSO-d_6_): δ (ppm) 57.8, 103.8, 114.5, 118.2, 120.1, 123.7, 126.4, 129.2, 130.2, 133.5, 133.8, 134.7, 135.2, 140.8, 153.7, 156.2, 159.8, 161.8. MS (EI, 20 eV) m/z (%): 384 (M+, 10), 239 (100). Anal. Calcd. For C_19_H_10_N_2_O_3_Cl_2_ (385.20) C, 59.22; H, 2.60; N, 7.27. Found: C, 59.12; H, 2.57; N, 7.13.

**2-Amino-5-oxo-4-(2,6-dichlorophenyl)-4H,5H-pyrano[3,2-c] chromene-3- carbonitrile (7g)**

White solid, mp = 257-259 ^o^C. Yield: (85%). IR (KBr) (*ν*_max_/cm^-1^) = 3475, 3326, 3247, 3158, 2246, 1720, 1692, 1654 cm^-1^. ^1^H NMR (500 MHz, DMSO-d_6_): δ (ppm) 5.08 (s, 1 H), 7.43 (d, ^3^*J* = 7.5 Hz, 1 H), 7.52 (d, ^3^*J* = 7.8 Hz, 1 H), 7.64 (br s, 2 H), 7.72 (d, ^3^*J* = 8.2 Hz, 1 H), 7.83 (t, ^3^*J* = 7.8 Hz, 1 H), 7.92 (d, ^3^*J* = 7.5 Hz, 1 H), 8.02 (t, ^3^*J* = 8.0 Hz, 1 H), 8.12 (d, ^3^*J* = 8.7 Hz, 1 H). ^13^C NMR (125 MHz, DMSO-d_6_): δ (ppm) 58.2, 104.3, 114.8, 118.6, 121.2, 124.7, 126.9, 129.8, 130.7, 133.6, 134.2, 134.7, 136.3, 141.7, 153.8, 156.4, 160.2, 161.8.

**12-(4-Chlorophenyl)-9,9-dimethyl-4-nitro-8,9,10,12-tetrahydro-11 H-benzo[a] xanthen-11-one (12a)**

White powder; mp 163-165 ^o^C; Yield: (92%). IR (KBr)(*ν*_max_/cm^-1^): 3125, 2963, 1674, 1595, 1387, 1375, 1276, 1254, 1129 cm^-1^. ^1^H NMR (500 MHz, DMSO-d_6_): δ (ppm) 0.87 (s, 3H), 1.07 (s, 3H), 2.14 (d, ^3^*J* = 15.8 Hz, 1H), 2.34 (d, ^3^*J* =15.8 Hz, 1H), 2.57 (d, ^3^*J* = 17.0 Hz, 1H), 2.67 (d,^3^*J* = 17.0 Hz, 1H), 4.87 (s, 1 H), 5.62 (s, 1H), 7.06 (d, ^3^*J* = 7.8 Hz, 1 H), 7.15 (d, ^3^*J* = 8.2 Hz, 2 H), 7.32 (d, ^3^*J* = 8.2 Hz, 2 H), 7.46 (t, ^3^*J* = 7.6 Hz, 1 H), 7.87 (d, ^3^*J* = 7.6 Hz, 1 H), 8.16 (d, ^3^*J* = 7.8 Hz, 1 H), 8.76 (d, ^3^*J* = 7.8 Hz, 1 H) ppm. ^13^C NMR (125 MHz, DMSO-d_6_): δ (ppm) 27.3, 29.8, 34.2, 36.8, 38.2, 52.0, 115.8, 116.5, 122.4, 127.3, 128.2, 129.4, 130.2, 130.8, 131.3, 132.2, 132.8, 133.4, 135.4, 149.6, 155.7, 164.3, 196.8 ppm. Anal. Calcd for C_25_H_20_ClNO_4_ (433.89) C, 69.21; H, 4.65; N, 3.23%. Found: C, 69.34; H, 4.76; N, 3.36.

**12-(3-Hydroxyphenyl)-3-methoxy-9,9-dimethyl-8,9,10,12-tetrahydro-11H-benzo[a] xanthen-11-one (12b)**

White powder; mp 163-165 ^o^C; Yield: (90%). IR (KBr)(*ν*_max_/cm^-1^): 3125, 2963, 1674, 1595, 1387, 1375, 1276, 1254, 1129 cm^-1^. ^1^H NMR (500 MHz, DMSO-d_6_): δ (ppm) 0.92 (s, 3H), 1.09 (s, 3H), 2.17 (d, ^3^*J* = 16.0 Hz, 1H), 2.36 (d, ^3^*J* =16.0 Hz, 1H), 2.58 (d, ^3^*J* = 16.8 Hz, 1H), 2.72 (d,^3^*J* = 16.8 Hz, 1H), 3.78 (s, 3 H), 4.88 (s, 1 H), 5.63 (s, 1H), 6.78 (s, 1 H), 7.02 (d, ^3^*J* = 7.6 Hz, 2 H), 7.12 (d, ^3^*J* = 7.6 Hz, 1 H), 7.14 (s, 1 H), 7.24 (t, ^3^*J* = 7.6 Hz, 1 H), 7.43 (d, ^3^*J* =7.8 Hz, 1 H), 7.65 (d, ^3^*J* = 7.6 Hz, 1 H), 7.75 (d, ^3^*J* = 7.8 Hz, 1 H) ppm. ^13^C NMR (125 MHz, DMSO-d_6_): δ (ppm) 27.2, 29.7, 33.2, 37.5, 38.6, 51.2, 53.4, 105.6, 114.2, 114.8, 117.4, 120.7, 123.5, 125.6, 126.3, 127.2, 128.4, 130.2, 132.0, 132.8, 138.7, 152.8, 154.6, 159.7, 168.2, 195.6 ppm. Anal. Calcd for C_26_H_24_O_4_ (400.47) C, 77.98; H, 6.04. Found: C, 78.14; H, 6.18. MS (EI, 20 eV): m/z (%) 400 (M^+^, 20), 323 (82), 77 (100).

**Ethyl 2-amino-8-bromo-5-oxo-4-phenyl-4H,5H-pyrano[3,2-c]chromene-3-carbonitrile (14a)**

Pale yellow solid, m.p. 263-265 ^o^C. Yield: (54%). IR (KBr) (*ν*_max_/cm^-1^) = 3365, 3278, 3178, 2163, 1727, 1673, 1629 cm^-1^. ^1^H NMR (500 MHz, DMSO-d_6_): δ (ppm) 1.23 (t, ^3^*J* = 7.3 Hz, 3 H), 4.23 (q, ^3^*J* = 7.3 Hz, 2 H), 4.68 (s, 1 H), 7.15 (t, ^3^*J* = 7.5 Hz, 2 H), 7.22 (t, ^3^*J* = 7.6 Hz, 1H), 7.38 (d, ^3^*J* = 7.7 Hz, 1 H), 7.62 (s, 1 H), 7.75 (d, ^3^*J* = 7.6 Hz, 2 H), 7.82 (d, ^3^*J* = 7.6 Hz, 1 H), 8.25 (br s, 2 H) ppm. ^13^C NMR (125 MHz, DMSO-d_6_): δ (ppm) 14.2, 41.6, 61.2, 79.8, 98.6, 113.7, 119.4, 125.2, 125.8, 126.8, 127.3, 128.6, 131.2, 148.6, 154.2, 157.2, 160.2, 162.3, 166.4 ppm. MS (EI, 20 eV): m/z (%) 443 (M^+^ +2, 15), 441 (M^+^, 15), 397 (68), 45 (100). Anal. Calcd for C_21_H_16_BrNO_5_ (442.26) C, 57.03; H, 3.65; N, 3.17. Found: C, 57.24; H, 3.74; N, 3.32.

**Ethyl 2-amino-9-methoxy-5-oxo-4-(4-bromophenyl)-4H,5H-pyrano[3,2-c] chromene-3-carbonitrile (14b)**

White solid, m.p. 272-274 ^o^C. Yield: (50%). IR (KBr) (*ν*_max_/cm^-1^) = 3412, 3367, 3246, 2254, 1732, 1692, 1585, 1275 cm^-1^. ^1^H NMR (500 MHz, DMSO-d_6_): δ (ppm) 1.28 (t, ^3^*J* = 7.4 Hz, 3 H), 3.75 (s, 3 H), 4.26 (q, ^3^*J* = 7.4 Hz, 2 H), 4.76 (s, 1 H), 7.16 (d, ^3^*J* = 7.6 Hz, 1 H), 7.23 (d, ^3^*J* = 7.8 Hz, 2 H), 7.32 (d, ^3^*J* = 7.5 Hz, 1 H), 7.38 (s, 1 H), 7.63 (d, ^3^*J* = 7.8 Hz, 2 H), 8.34 (br s, 2 H) ppm. ^13^C NMR (125 MHz, DMSO-d_6_): δ (ppm) 14.3, 42.3, 55.6, 61.5, 80.2, 99.3, 111.2, 113.7, 115.7, 116.5, 120.2, 128.3, 135.2, 146.5, 148.6, 144.2, 157.3, 160.2, 162.3, 165.4 ppm. MS (EI, 20 eV): m/z (%) 473 (M^+^ +2, 14), 471 (M^+^, 14), 427 (78), 45 (100). Anal. Calcd for C_22_H_18_BrNO_6_ (472.29) C, 55.95; H, 3.84; N, 2.97. Found: C, 56.12; H, 3.94; N, 3.15.

**8-bromo-2,5-dioxo-4-phenyl-3,4-dihydro-2H,5H-pyrano[3,2-c]chromene-3-carbonitrile (15a)**

White solid, m.p. 226-228 ^o^C. Yield: (54%). IR (KBr) (*ν*_max_/cm^-1^) = 2154, 1736, 1695, 1592, 1478, 1325 cm^-1^. ^1^H NMR (500 MHz, DMSO-d_6_): δ (ppm) 3.48 (d, ^3^*J* = 6.8 Hz, 1H), 4.47 (d, ^3^*J* = 6.8 Hz, 1H), 7.16 (t, ^3^*J* = 7.6 Hz, 2 H), 7.35 (t, ^3^*J* = 7.6 Hz, 1H), 7.42 (d, ^3^*J* = 7.7 Hz, 1 H), 7.56 (s, 1 H), 7.82 (d, ^3^*J* = 7.7 Hz, 1 H), 8.02 (d, ^3^*J* = 7.6 Hz, 2 H) ppm. ^13^C NMR (125 MHz, DMSO-d_6_): δ (ppm) 39.2, 51.6, 106.5, 112.2, 116.3, 118.6, 123.4, 125.6, 126.2, 126.9, 127.5, 128.4, 136.2, 154.8, 158.7, 160.2, 161.4 ppm. MS (EI, 20 eV): m/z (%) 397 (M^+^ +2, 15), 395 (M^+^, 15), 319 (62), 77 (100). Anal. Calcd for C_19_H_10_BrNO_4_ (396.19) C, 57.60; H, 2.54; N, 3.54. Found: C, 57.72; H, 2.65; N, 3.63.

**4-(4-bromophenyl)-9-methoxy-2,5-dioxo-3,4-dihydro-2H,5H-pyrano[3,2-c]chromene-3-carbonitrile (15b)**

White solid, m.p. 268-270 ^o^C. Yield: (50%). IR (KBr) (*ν*_max_/cm^-1^) = 2158, 1738, 1698, 1587, 1464, 1356 cm^-1^. ^1^H NMR (500 MHz, DMSO-d_6_): δ (ppm) 3.42 (d, ^3^*J* = 6.5 Hz, 1H), 3.75 (s, 3H), 4.45 (d, ^3^*J* = 6.5 Hz, 1H), 7.15 (d, ^3^*J* = 7.8 Hz, 1 H), 7.28 (d, ^3^*J* = 7.8 Hz, 1 H), 7.38 (d, ^3^*J* = 7.9 Hz, 2 H), 7.45 (s, 1 H), 7.96 (d, ^3^*J* = 7.9 Hz, 2 H) ppm. ^13^C NMR (125 MHz, DMSO-d_6_): δ (ppm) 39.4, 51.5, 55.6, 106.7, 112.3, 112.8, 114.2, 116.3, 117.2, 123.5, 130.2, 131.4, 134.2, 148.3, 151.8, 158.6, 161.4, 162.3 ppm. MS (EI, 20 eV): m/z (%) 427 (M^+^ +2, 8), 425 (M^+^, 8), 395 (78), 156 (48), 31 (100). Anal. Calcd for C_20_H_12_BrNO_5_ (426.22) C, 56.36; H, 2.84; N, 3.29. Found: C, 56.43; H, 2.92; N, 3.38.

**4a spectra**

**
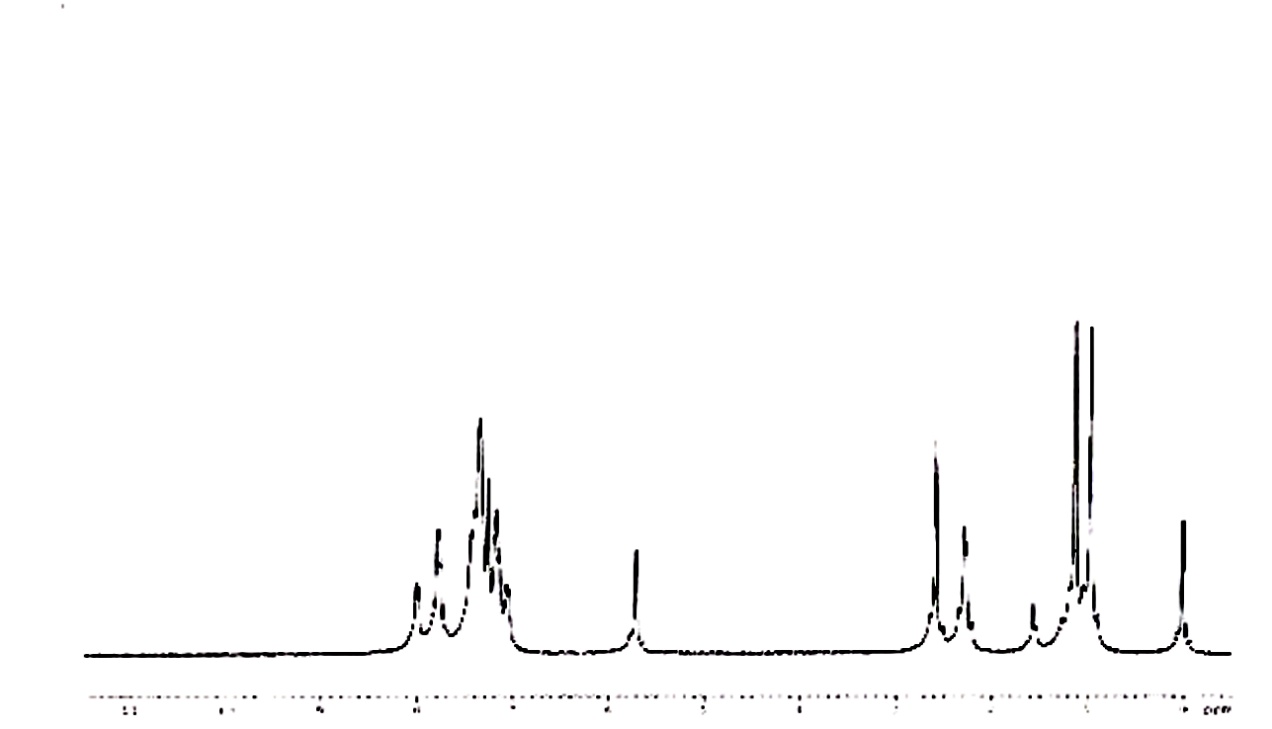
**

**4b spectra**

**
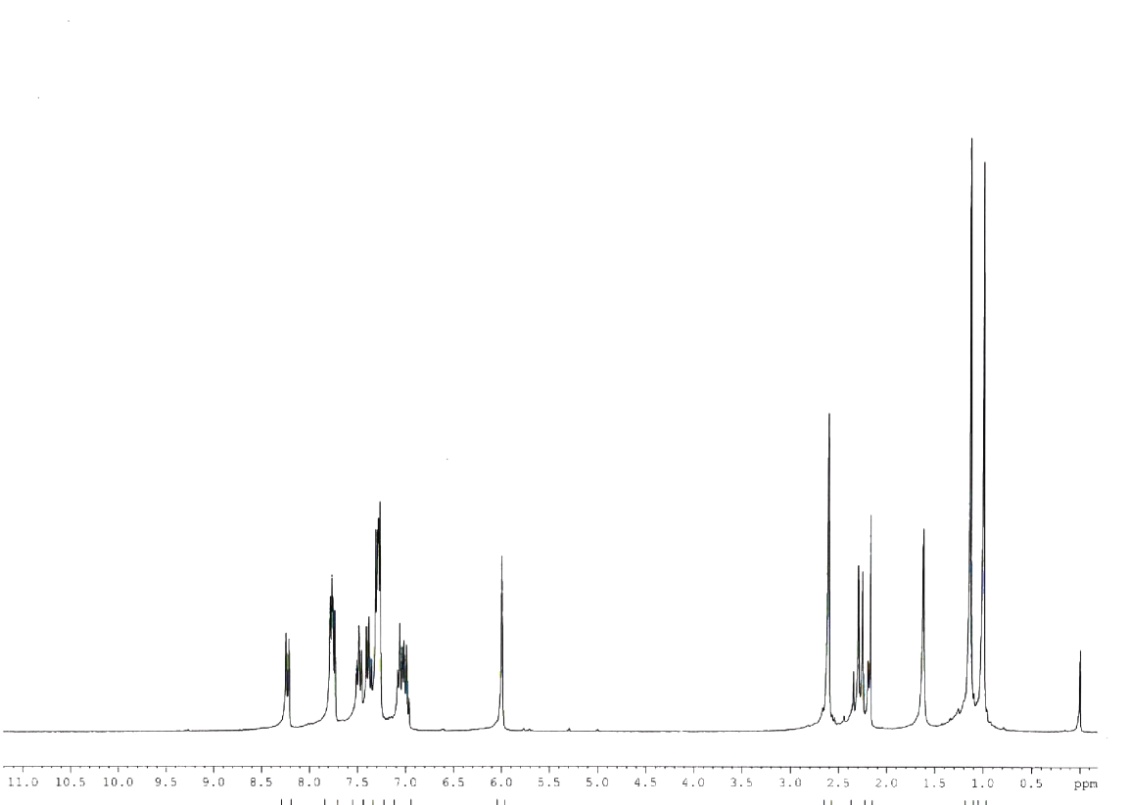
**

**4c spectra**


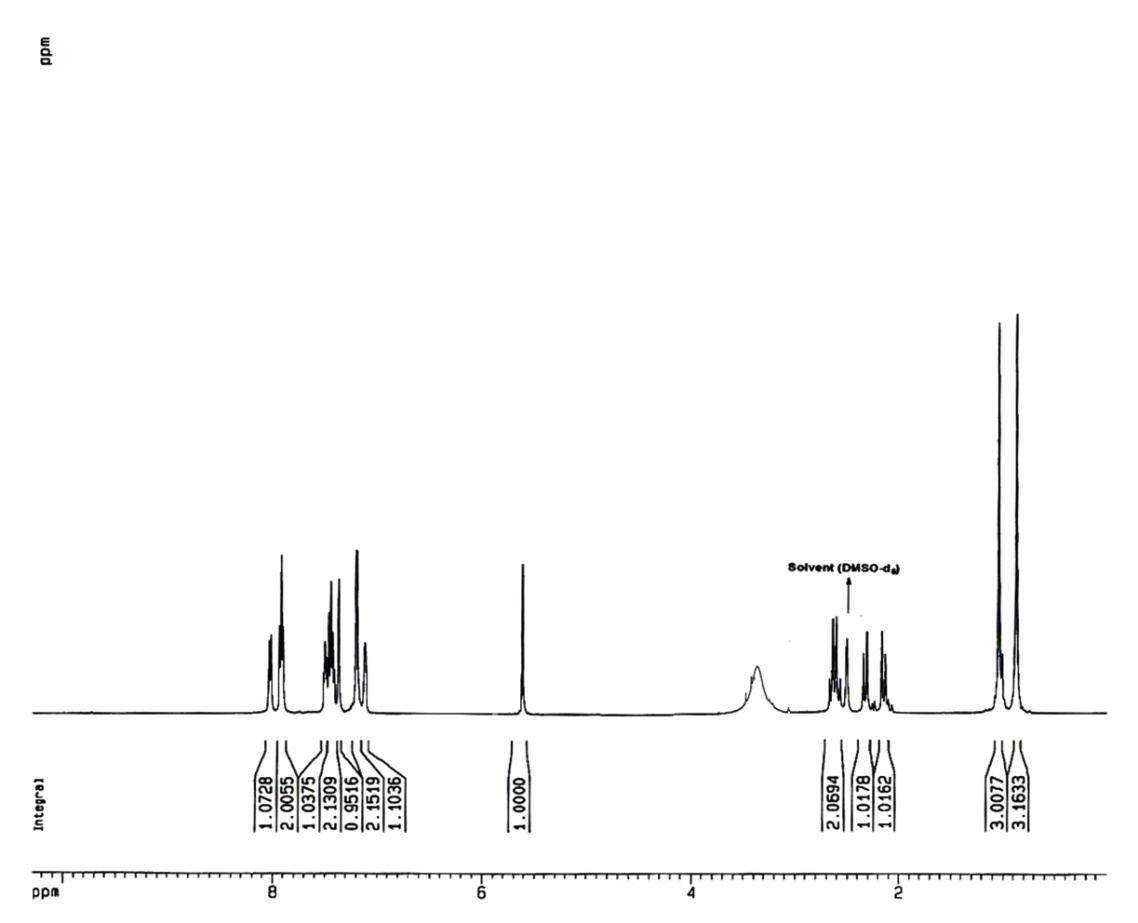


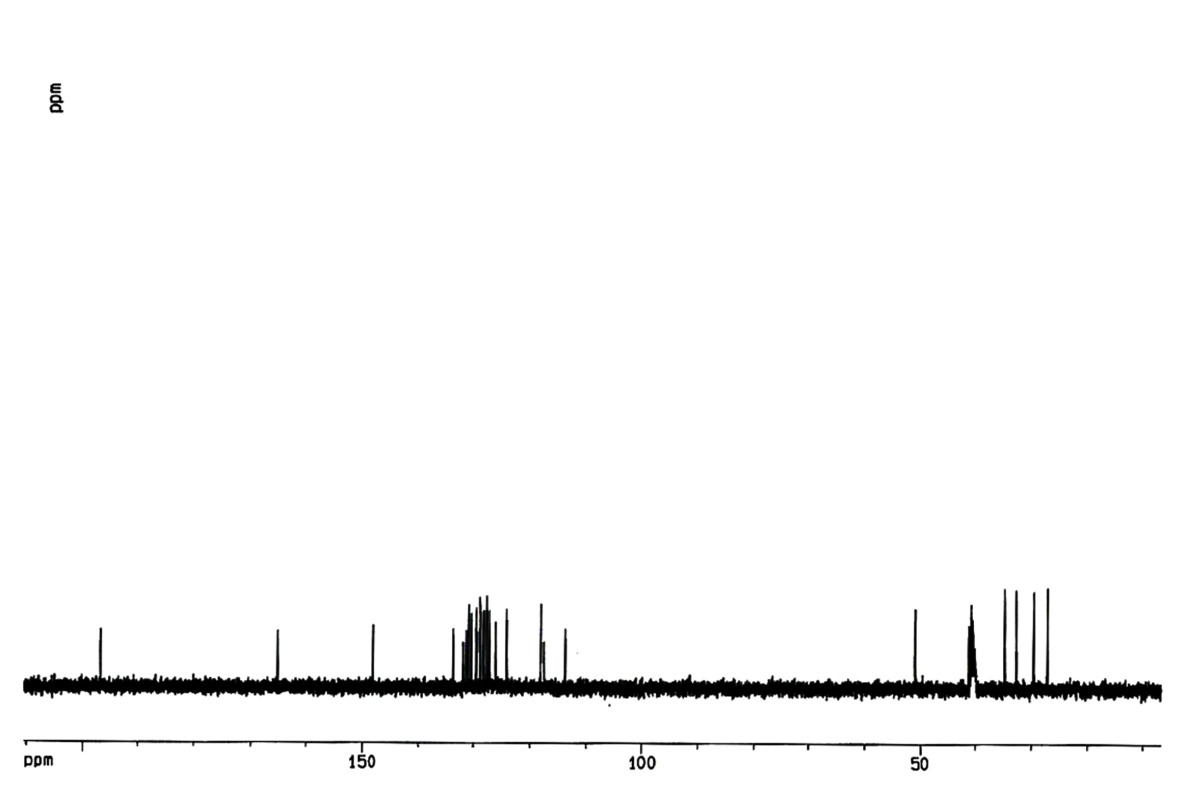


**4d spectra**

**
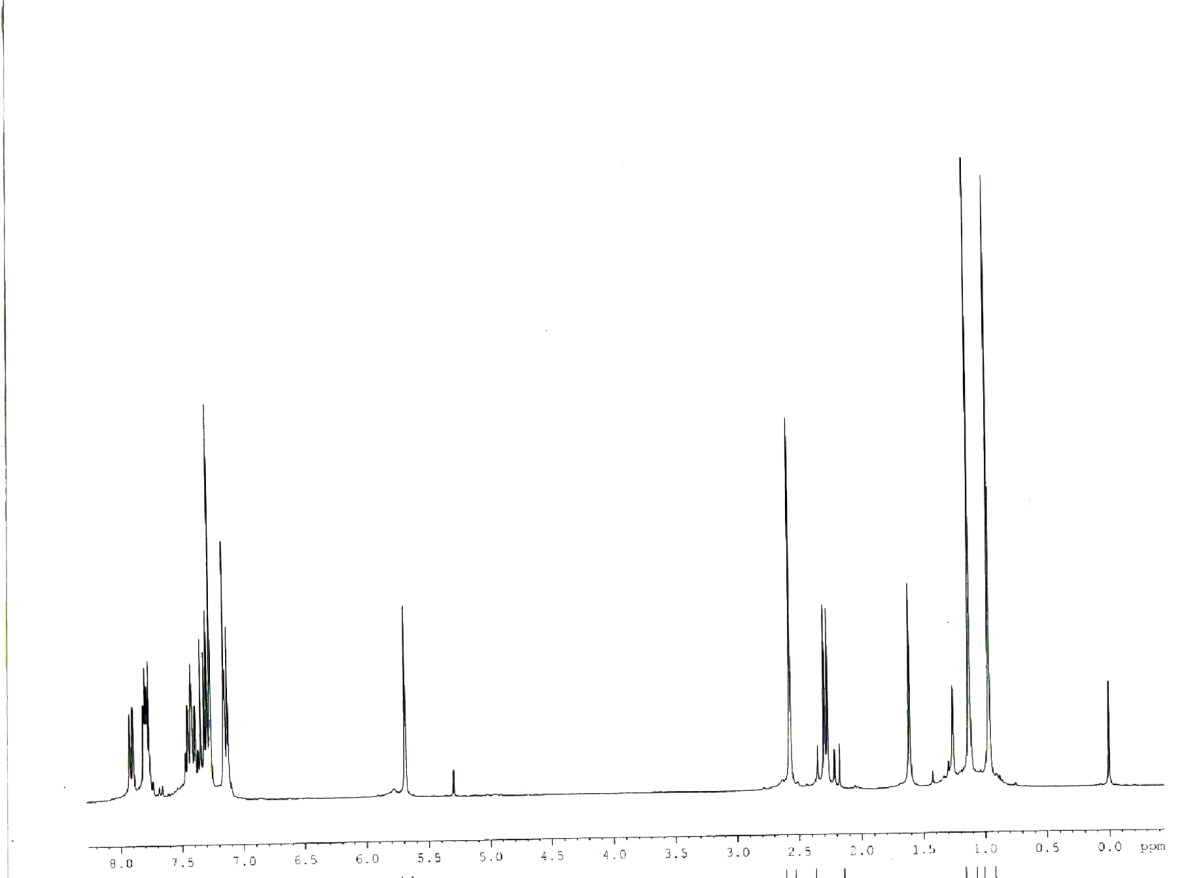
**

**4e spectra**


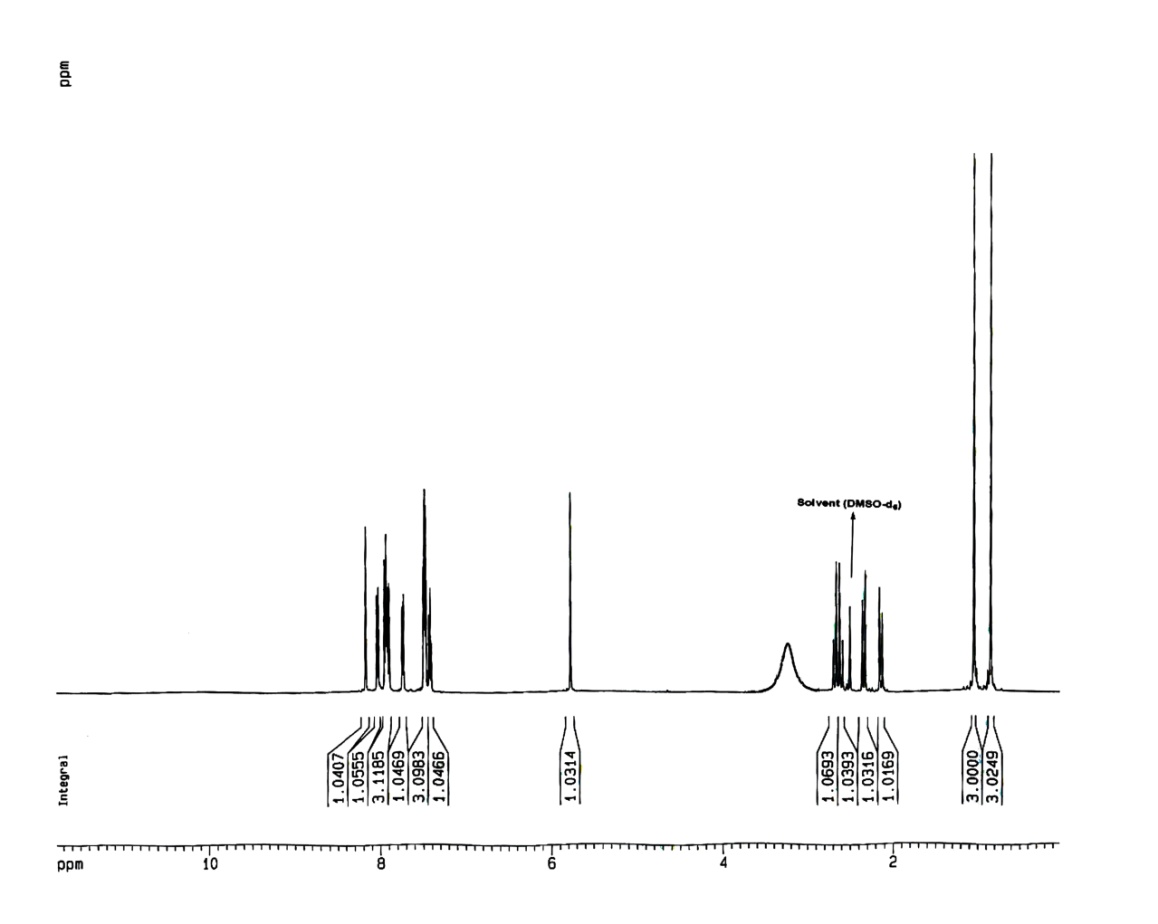


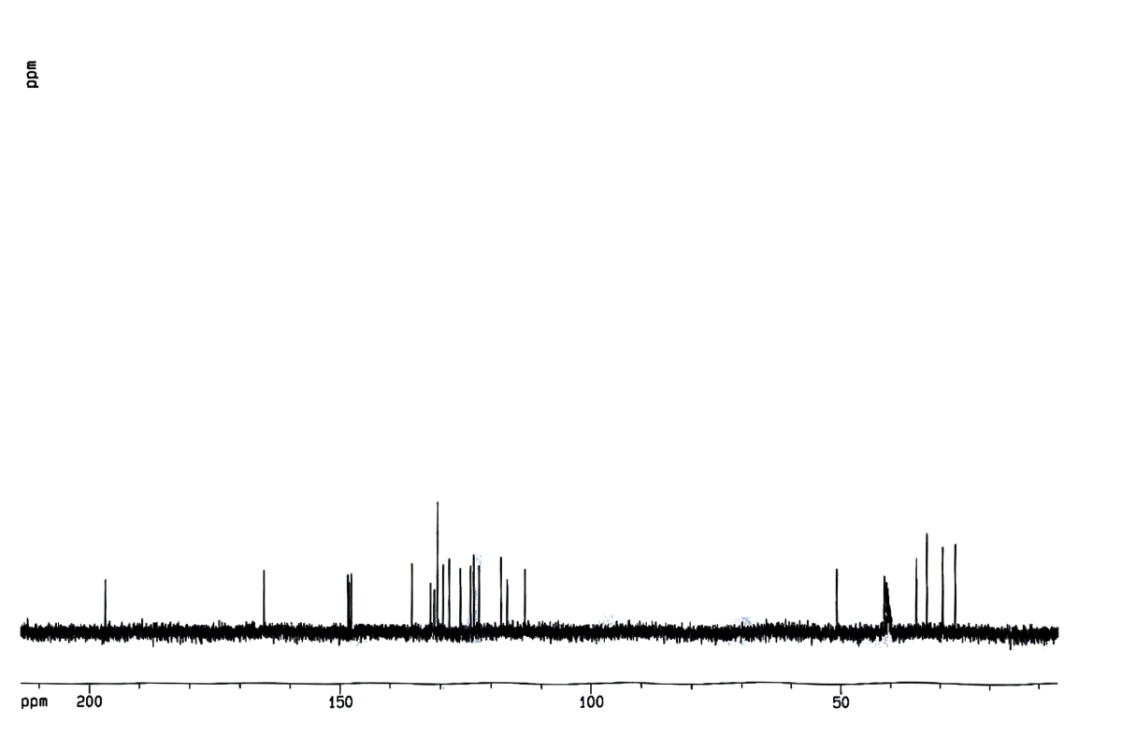


**4fspectra**


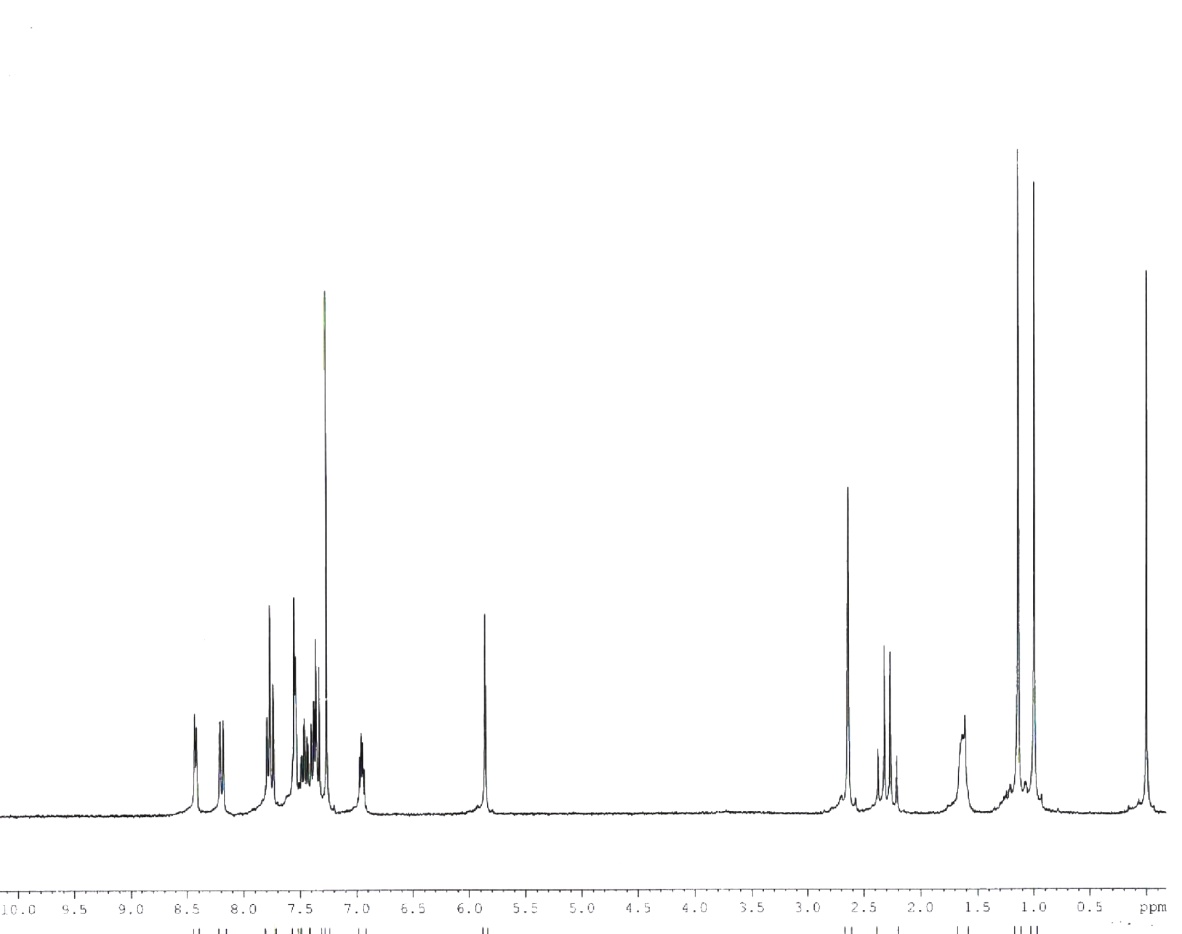


**4gspectra**

**
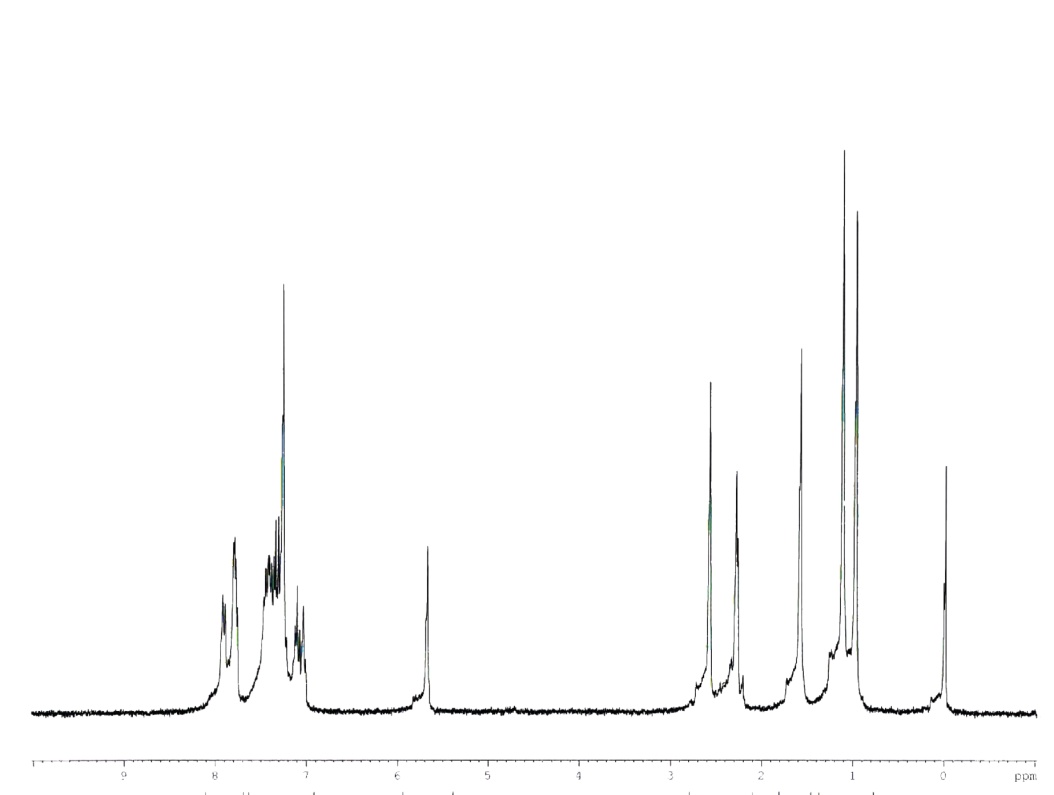
**

**4hspectra**


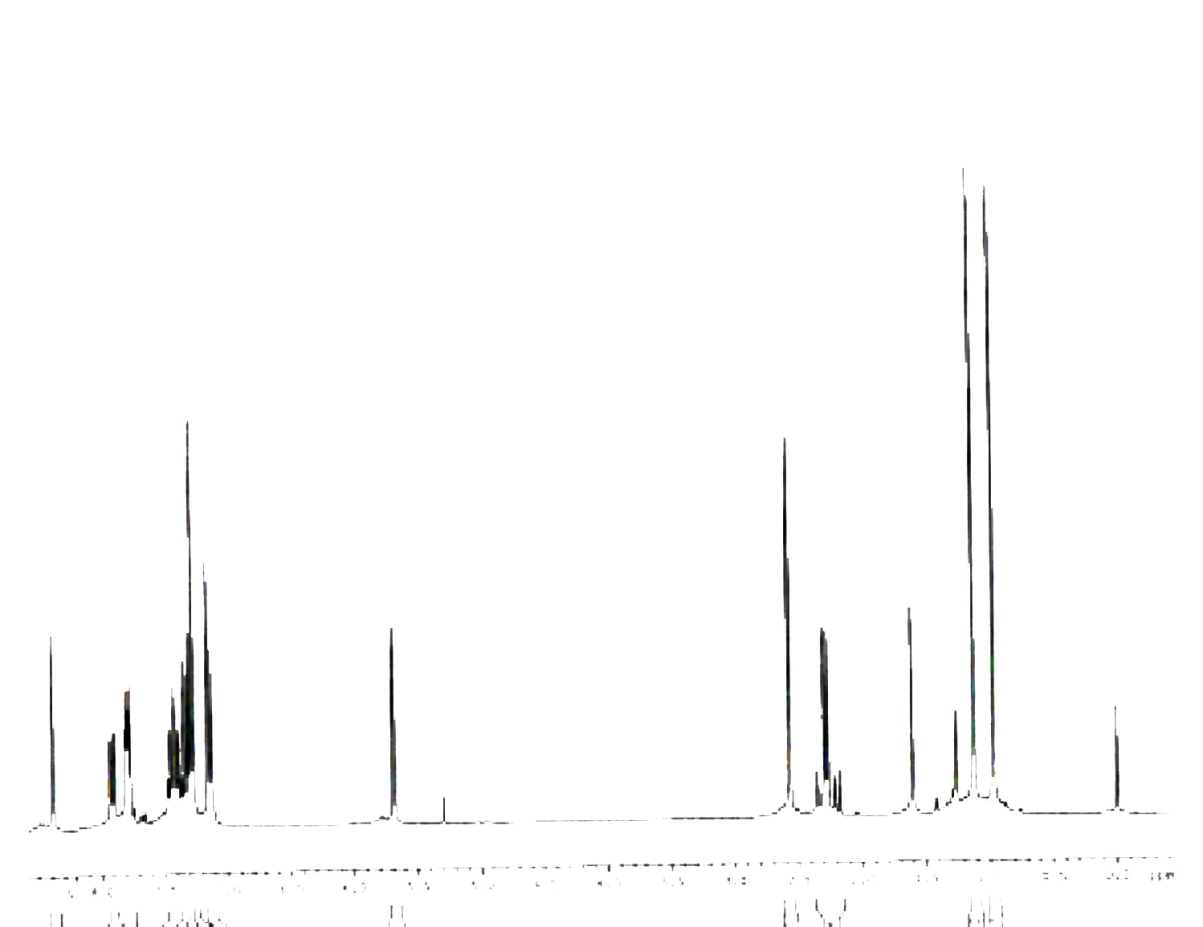


**4ispectra**


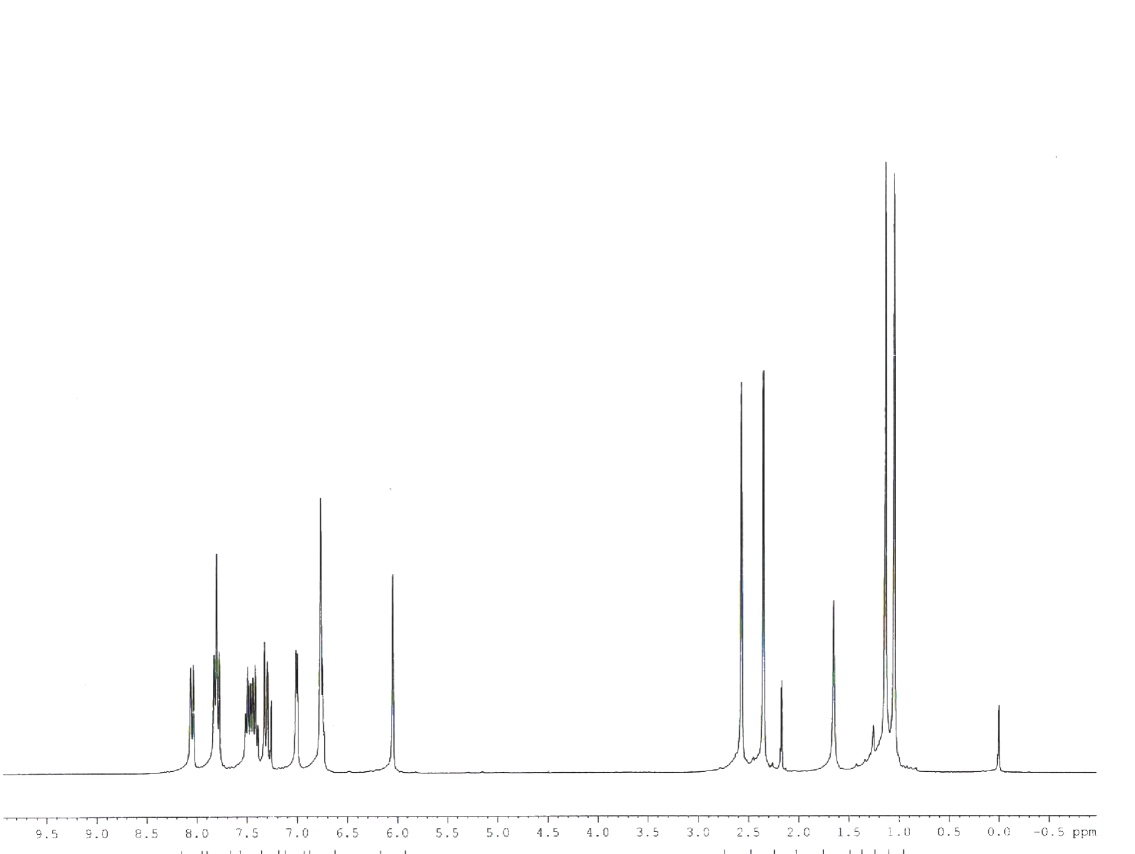


**7a spectra**


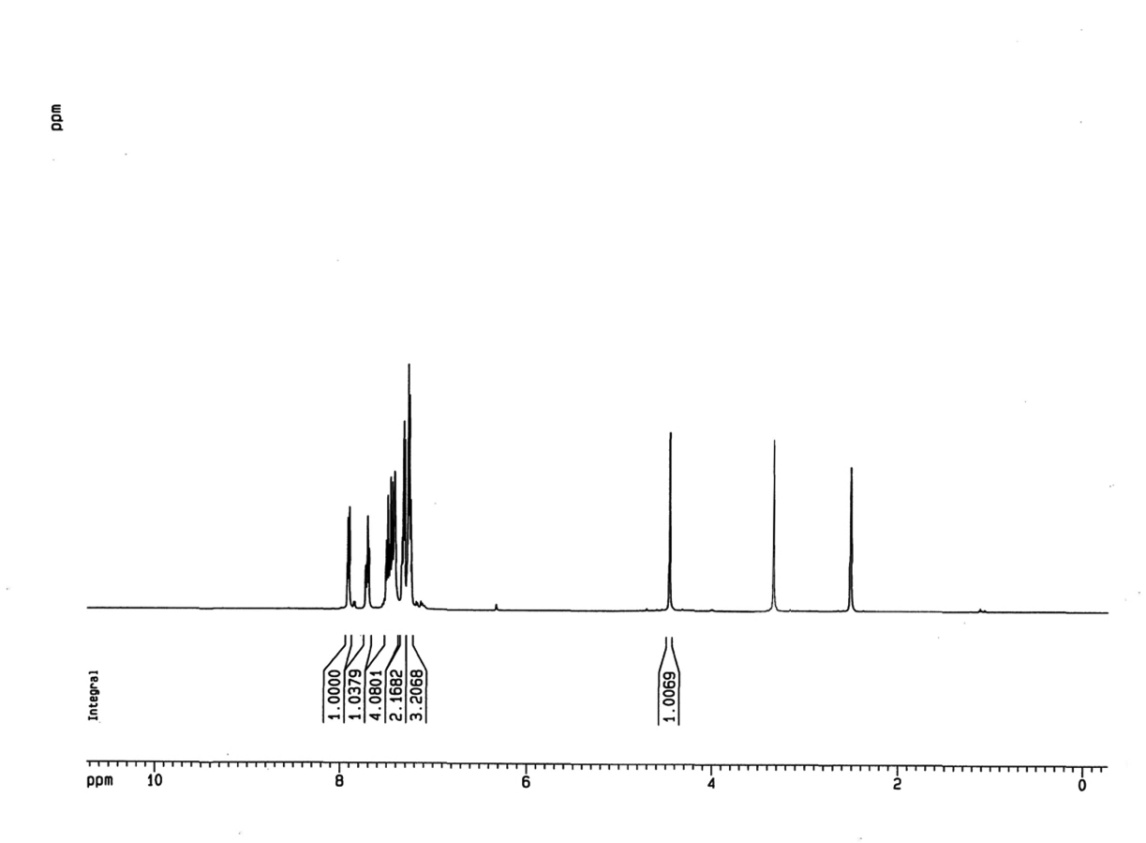


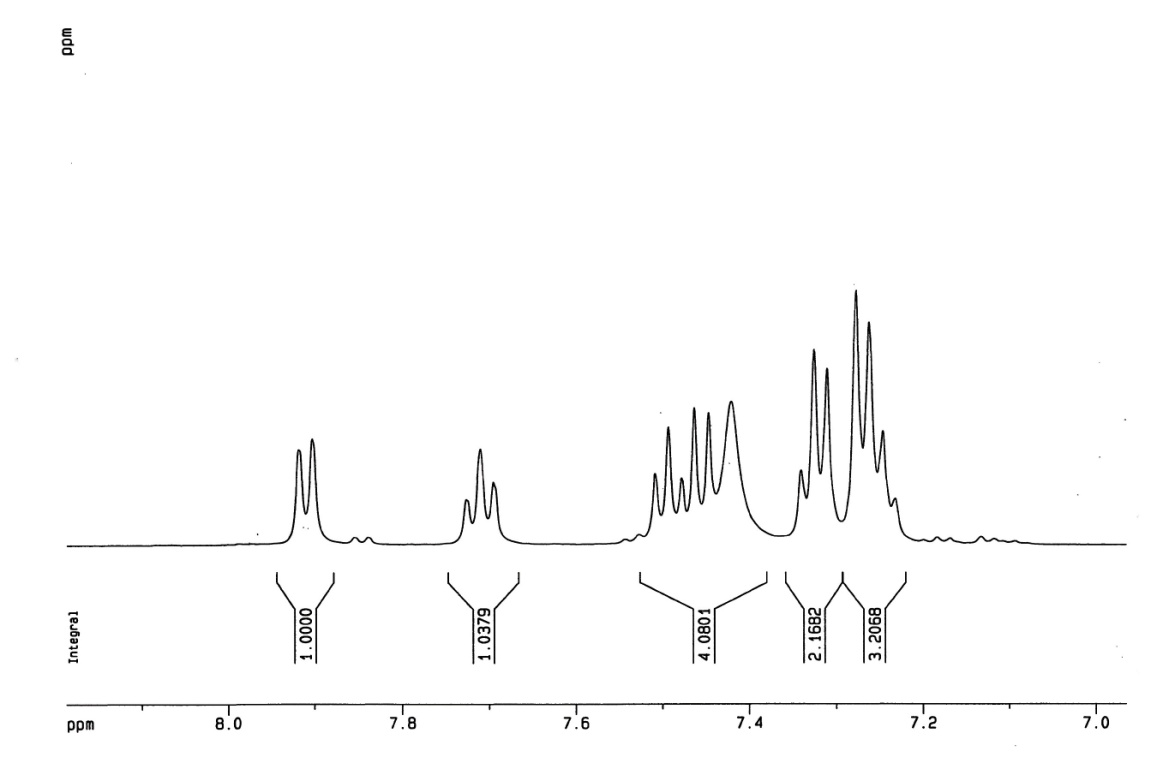


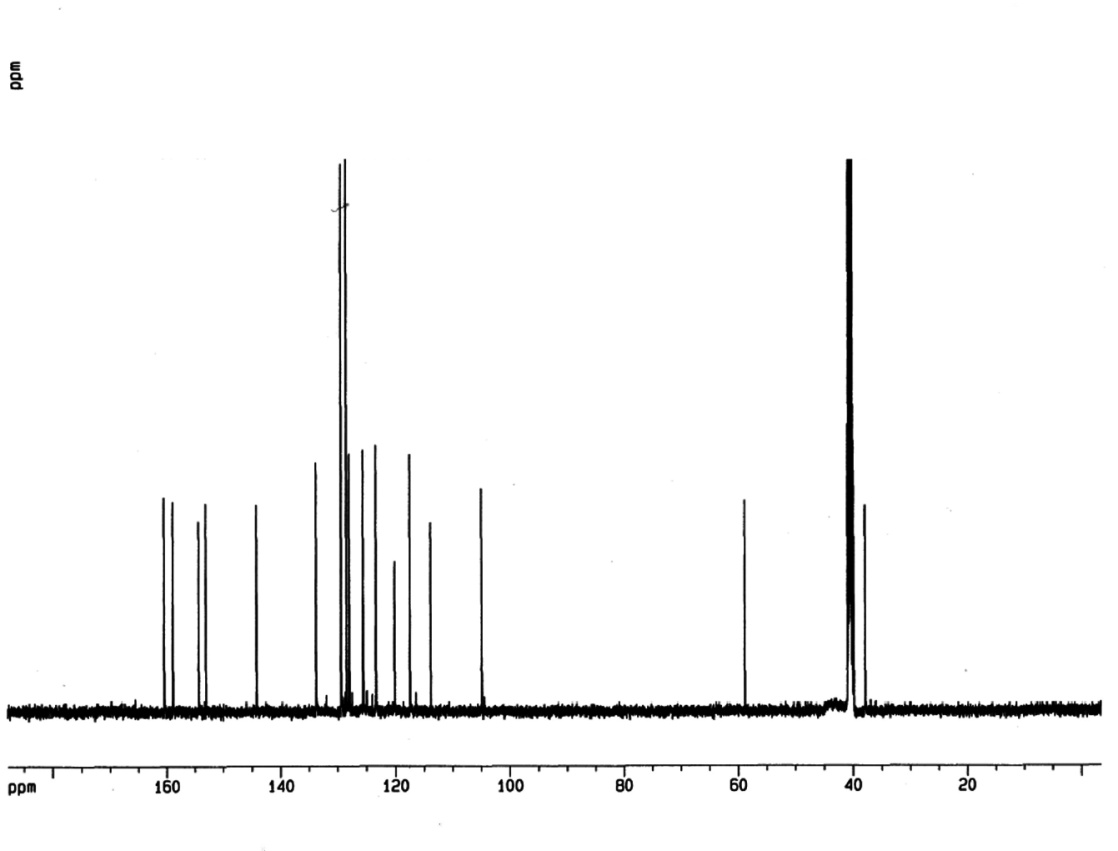


**7b spectra**

**
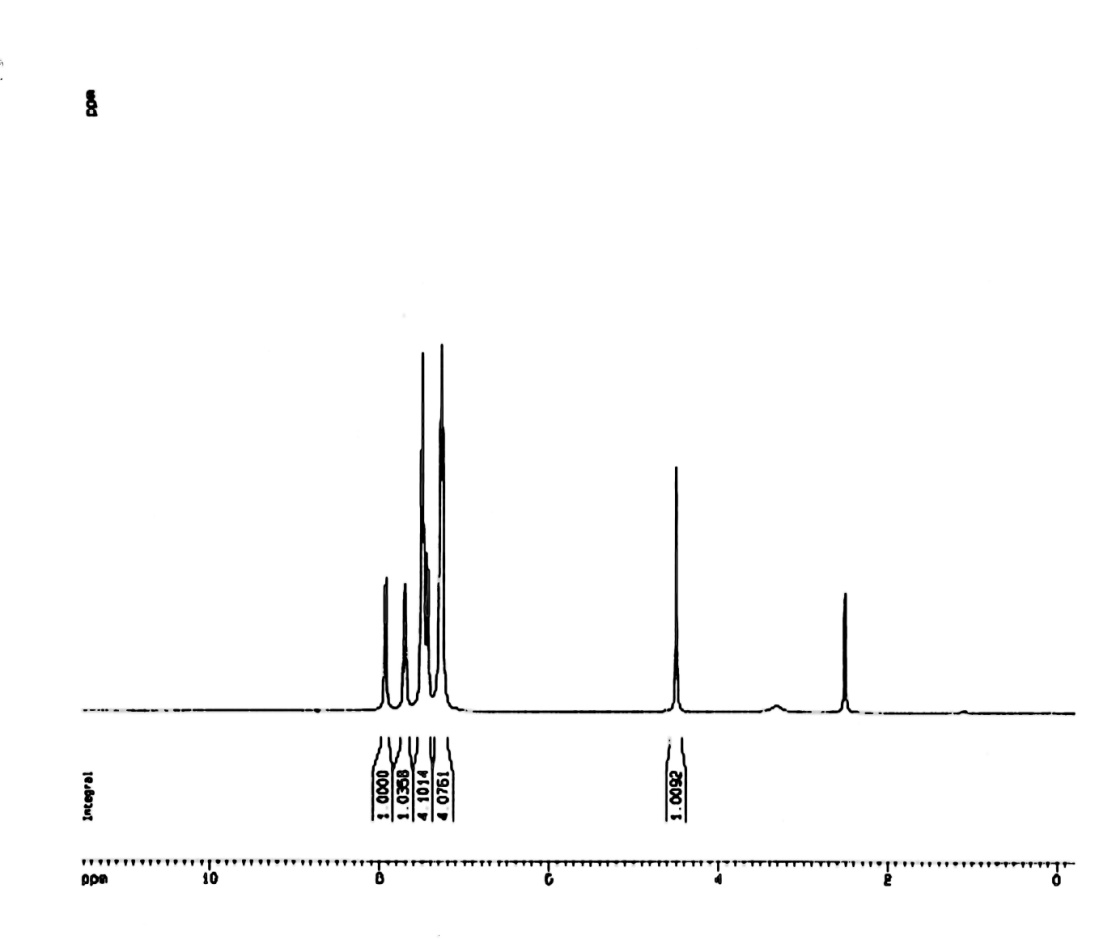
**

**
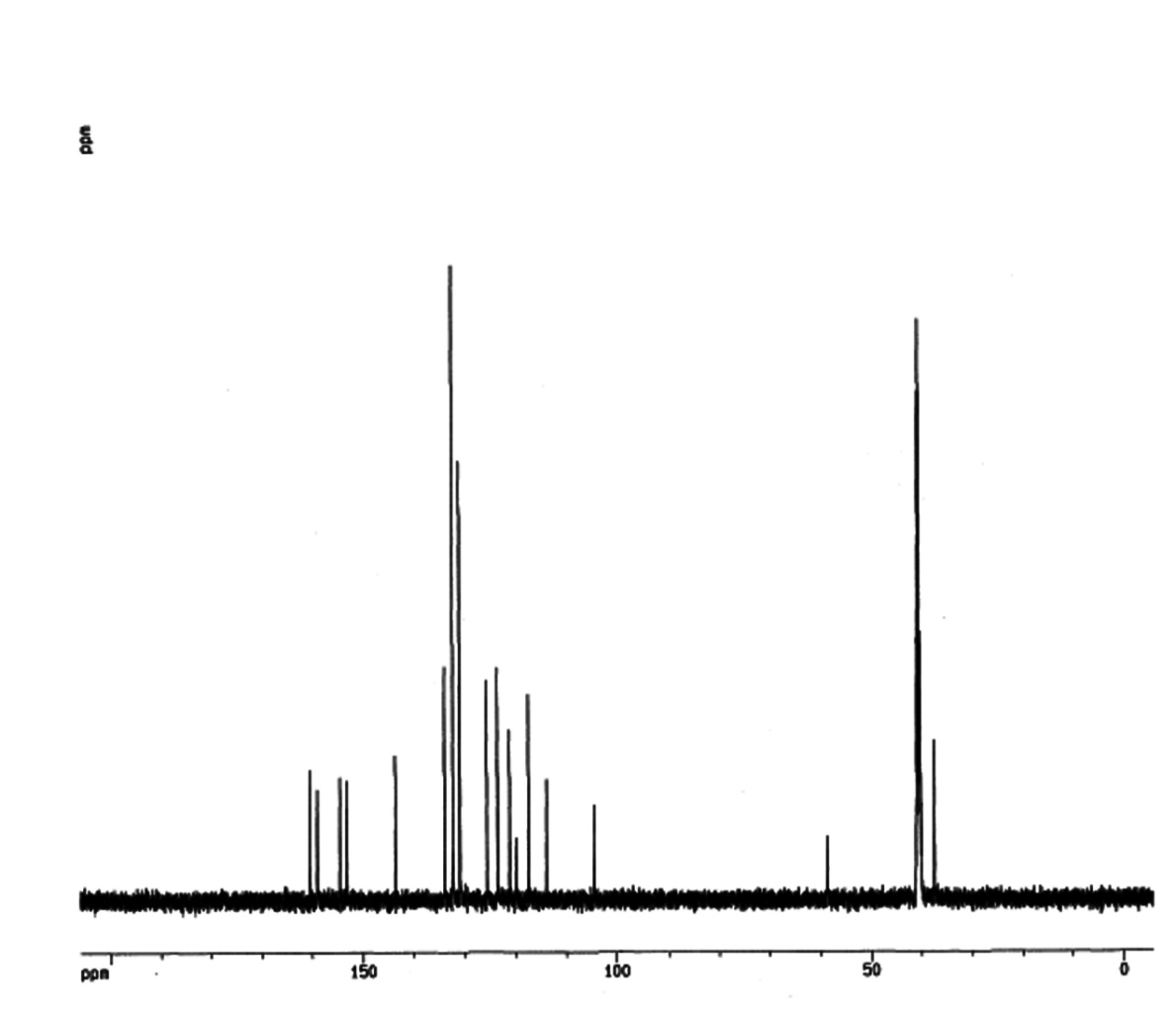
**

**7c spectra**

**
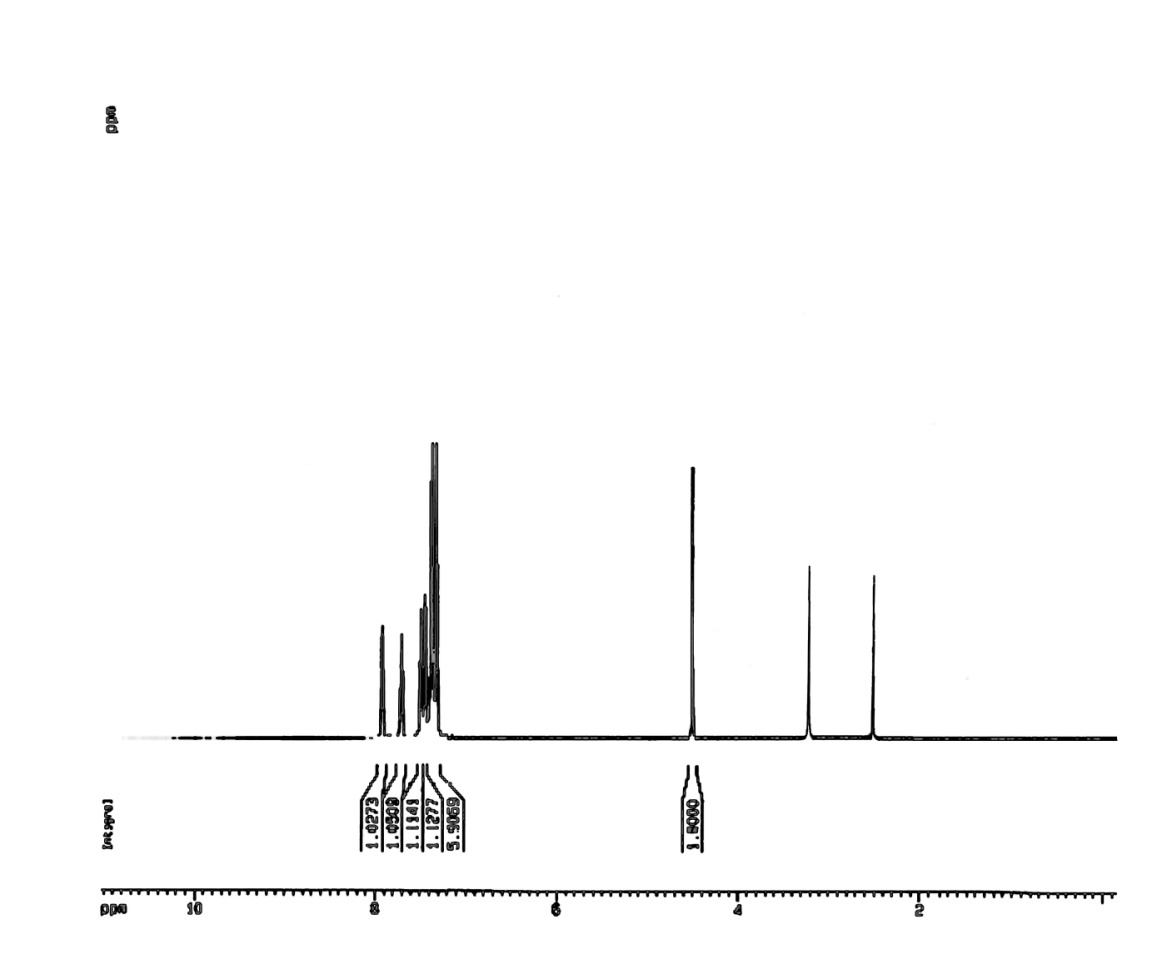
**

**
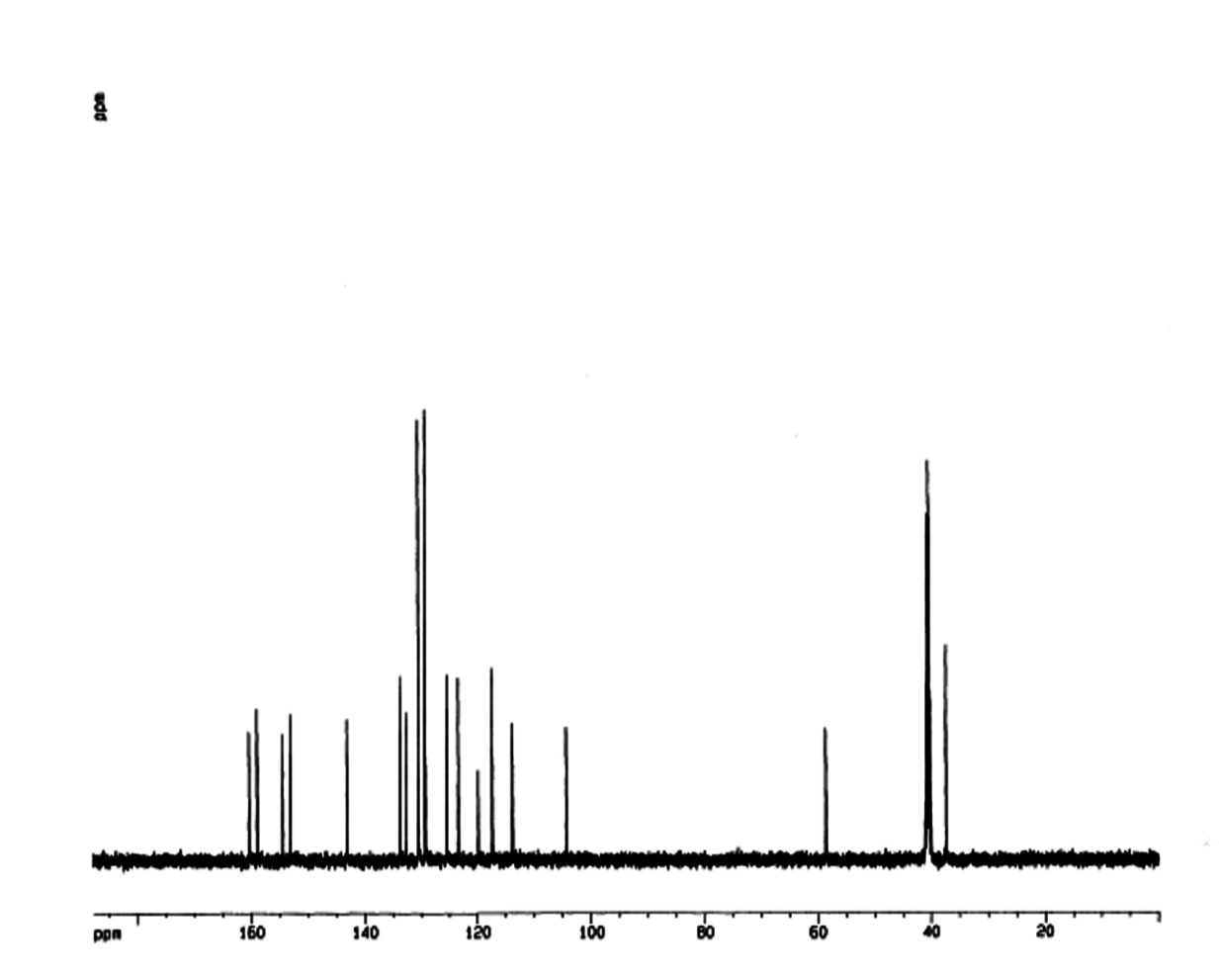
**

**7d spectra**

**
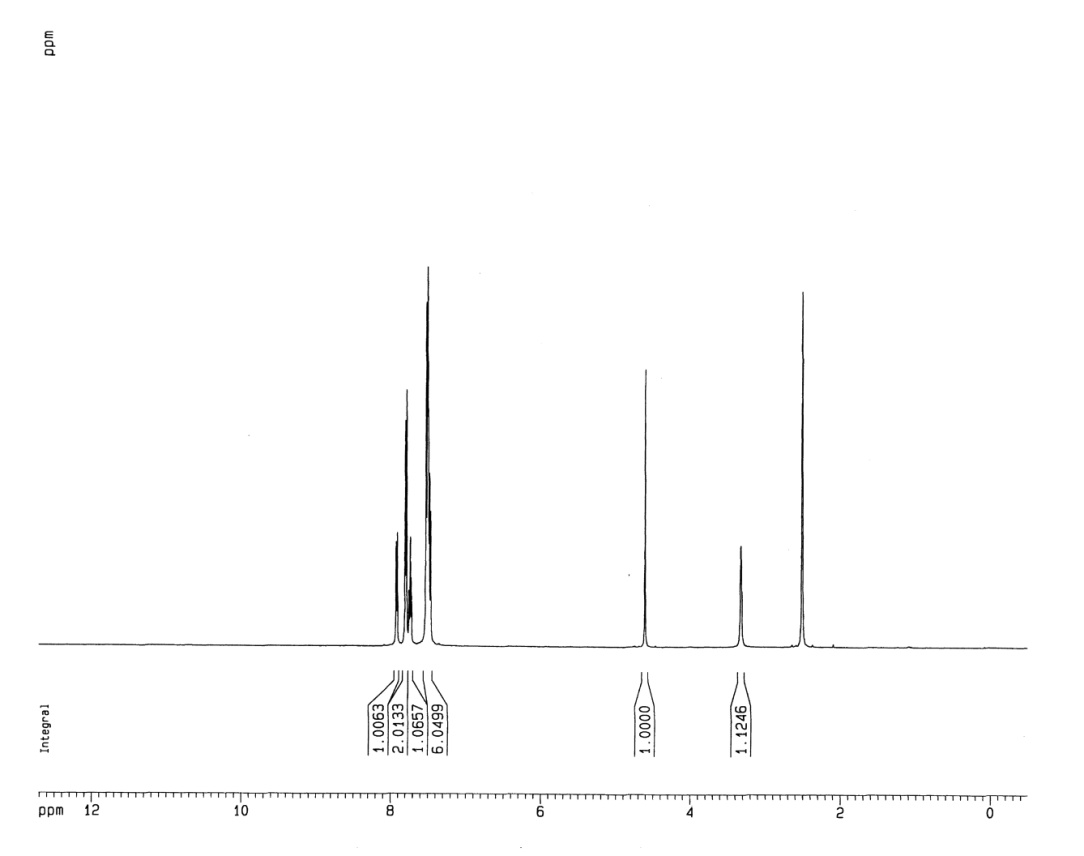
**

**7e spectra**

**
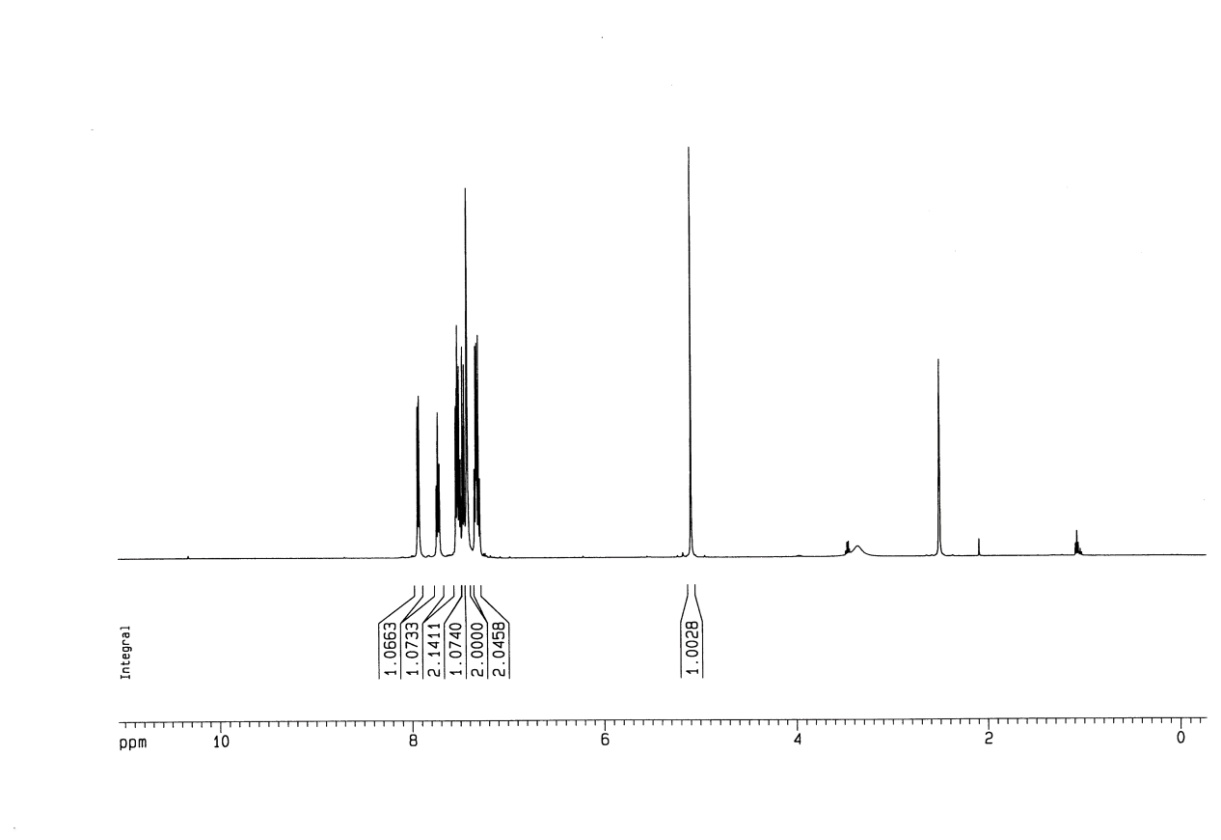
**

**7f spectra**

**
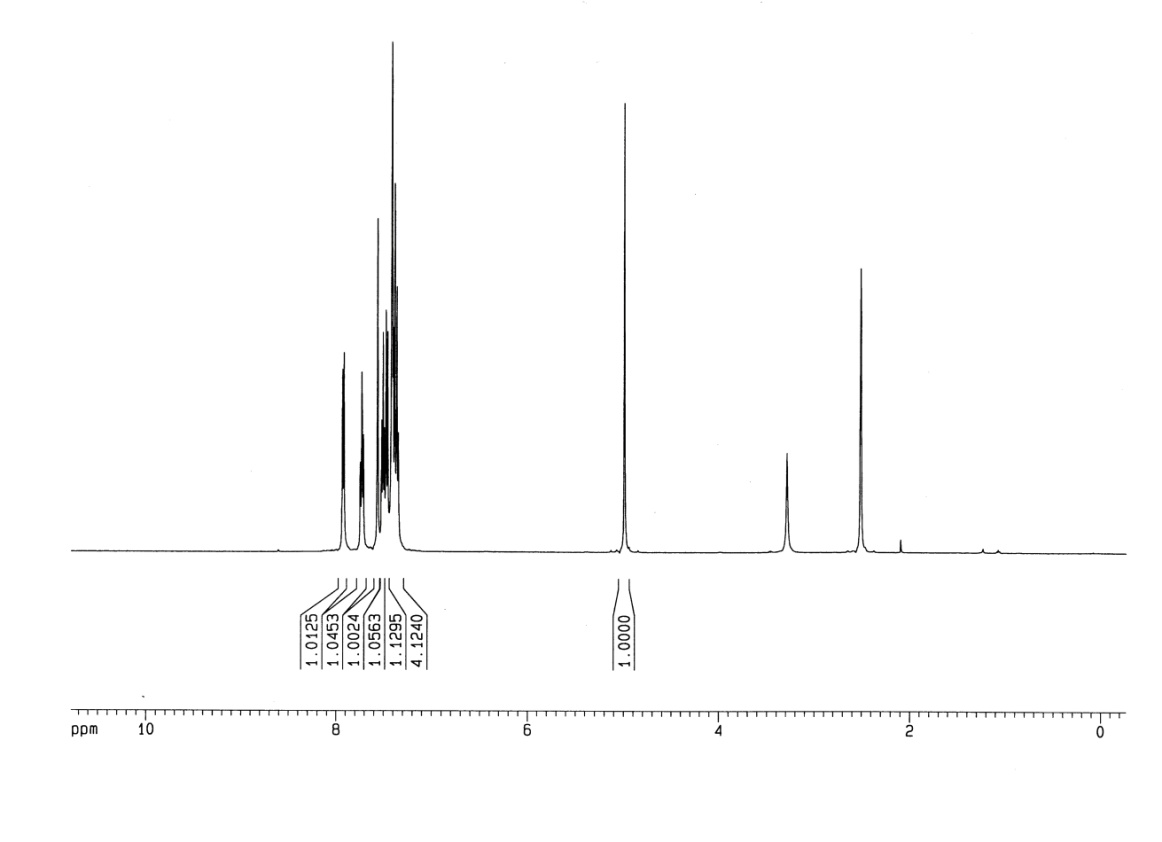
**

**7g spectra**

**
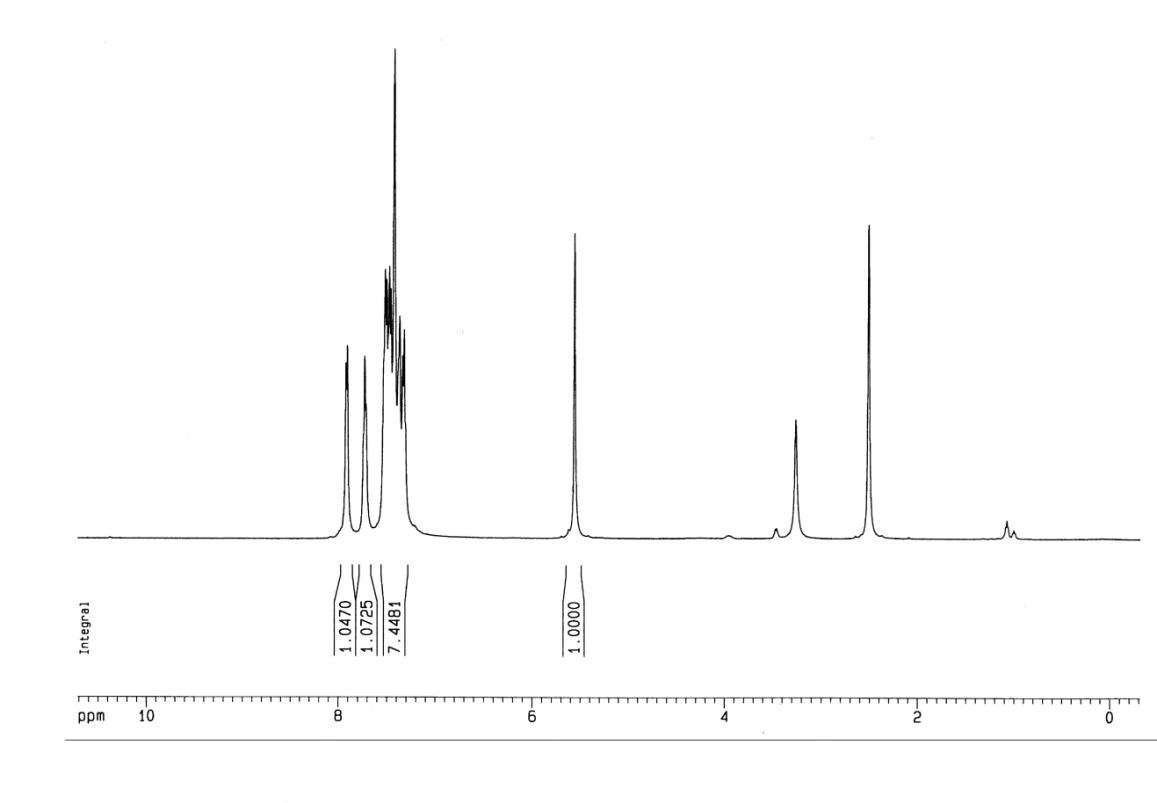
**

**12a spectra**

**
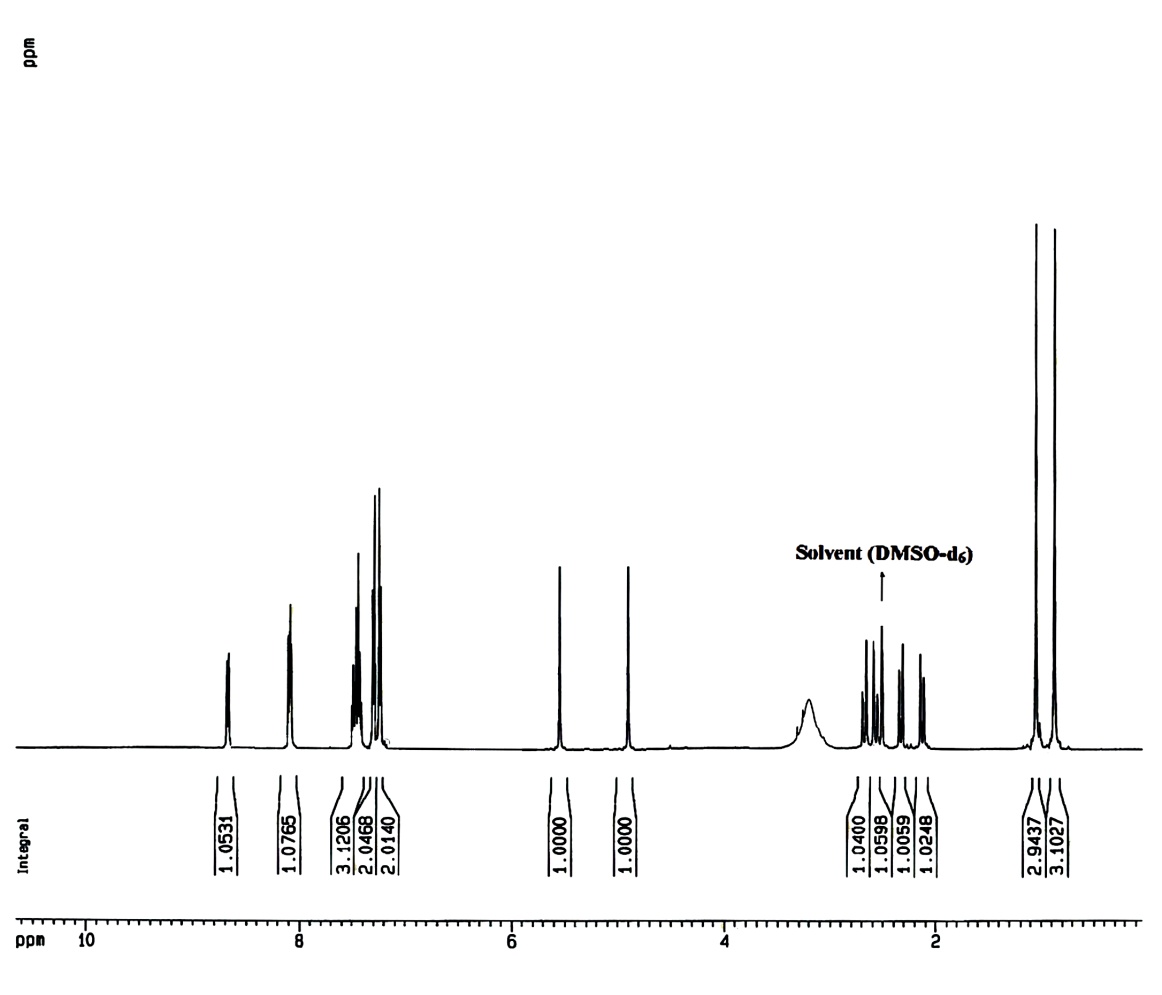
**

**
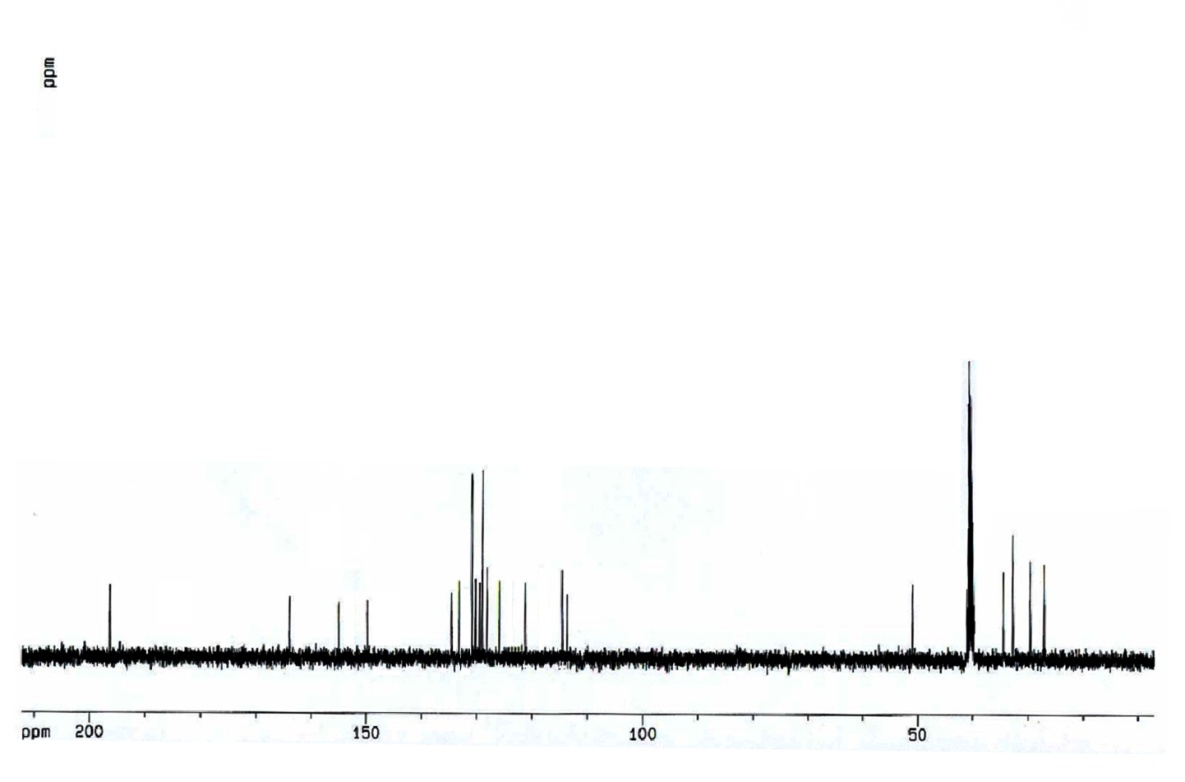
**
